# Potential anti‐obesity effect of saponin metabolites from adzuki beans: A computational approach

**DOI:** 10.1002/fsn3.4032

**Published:** 2024-02-22

**Authors:** Ashaimaa Y. Moussa, Abdullah Alanzi, Jinhai Luo, Sookja Kim Chung, Baojun Xu

**Affiliations:** ^1^ Department of Pharmacognosy, Faculty of Pharmacy Ain Shams University Cairo Egypt; ^2^ Department of Pharmacognosy, College of Pharmacy King Saud University Riyadh Saudi Arabia; ^3^ Department of Life Sciences, Food Science and Technology Program BNU‐HKBU United International College Zhuhai Guangdong China; ^4^ Medical Faculty Macau University of Science and Technology Macau China

**Keywords:** aduzki bean, enzyme, molecular docking, molecular simulation, obesity, saponin

## Abstract

In contrast to its widespread traditional and popular culinary use to reduce weight, *Vigna angularis* (adzuki beans) was not subjected to sufficient scientific scrutiny. Particularly, its saponins whose role was never investigated before to unveil the beans’ antidiabetic and anti‐obesity effects. Four vital pancreatic and intestinal carbohydrate enzymes were selected to assess the potency of the triterpenoidal saponins of *V. angularis* to bind and activate these proteins through high‐precision molecular modeling and dynamics mechanisms with accurate molecular mechanics Generalized Born Surface Area (MMGBSA) energy calculations; thus, recognizing their anti‐obesity potential. Our results showed that adzukisaponin VI and adzukisaponin IV were the best compounds in the *α*‐amylase and *α*‐glucosidase enzymatic grooves, respectively. Adzukisaponin VI and angulasaponin C were the best fitting in the N‐termini of sucrase‐isomaltose (SI) enzyme, and angulasaponin C was the best scoring compound in maltase‐glucoamylase C‐termini. All of them outperformed the standard drug acarbose. These compounds in their protein complexes were selected to undergo molecular simulations of the drug‐bound protein compared to the apo‐protein through 100 ns, which confirmed the consistency of binding to the key amino acid residues in the four enzyme pockets with the least propensity of unfolding. Detailed analysis is given of the different polar and hydrophobic binding interactions of docked compounds. While maltase–adzukisaponin VI complex scored the lowest MMGBSA free energy of −67.77 Kcal/mol, α‐amylase complex with angulasaponin B revealed the free binding energy of −74.18 Kcal/mol with a dominance of van der Waals energy (ΔEVDW) and the least change from the start to the end of the simulation time. This study will direct researchers to the significance of isolating the pure adzuki saponin components to conduct future in vitro and in vivo experimental works and even clinical trials.

## Graphical abstract

Adzukisaponin VI, adzukisaponin IV, and angulasaponins B and C were the best compounds in all enzymatic grooves. Molecular dynamics (MD) simulation and free energy molecular mechanics Generalized Born Surface Area (MMGBSA) calculations manifested stability of binding and absence of unfolding in the best scoring complexes. Saponins’ bioactivity rather than polyphenolics could rationalize the anti‐obesity effect of adzuki beans.

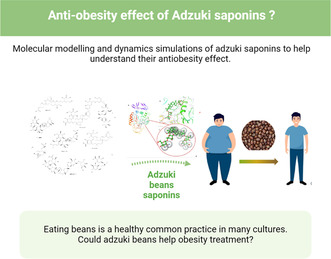

## 1 INTRODUCTION

Natural products have been the source of bioactive molecules since antiquity with marked effectiveness against various ailments, such as inflammation, cancer, infection, bone diseases, and more (Ashraf et al., 2020, 2022; Moussa et al., 2020; Fayez et al., 2022; Moussa, 2013). In Japan, *V. angularis*, known as adzuki beans, were considered a part of the traditional Kampo medicine, which prescribed its juice and decoction to reduce aging and cardiovascular diseases (CVDs) (Maruyama et al., 2008). Eating beans to lose weight is a common folk practice in many countries, such as China, Egypt, Middle Eastern region, Japan, and South Korea*. Vigna angularis*, or Chi Xiao dou in Chinese, were used in the treatment of dropsy and beriberi diseases in China and even mentioned in the first medical herbal book in the sixteenth century, the Ben Cao Gang Mu (Compendium of Materia Medica) (Jiang et al., 2014; Wang et al., 2022). Obesity‐related diseases can dramatically retard healthcare efforts and incur extra costs on governments, which renders safe and effective anti‐obesity solutions an urgent need. Saponins were never thought before to be of a significant role in adzuki beans’ favorable effect in diabetes and glucose intolerance (Shi et al., 2020), and adzuki studies about obesity and weight loss were very few despite the popular use, which necessitated scientific work to rationalize these notable health benefits (Liu et al., 2017). Reports included reduction of total cholesterol levels, triglycerides, hepatic cholesterol, and low‐density lipoprotein (LDL) cholesterol by the virtue of pancreatic lipase and α‐glucosidase inhibitory effects of adzuki beans flavonoids. Our previous work (Liu et al., 2017) identified through high‐performance liquid chromatography–diode array detection–electrospray ionization–mass spectrometry analyses (HPLC–DAD–ESI–MS) four flavonoids and six oleanane‐type triterpene oligoglycosides named adzukisaponins in the ethanolic extracts of the red beans (Liu et al., 2017). Most of the isolated saponins reported an inhibition of nitric oxide (NO) production (Sansbury & Hill, 2014), which was proved to be closely correlated with the anti‐obesity mechanism (Qian et al., 2019) (Table 1). Yet, activities on pure compounds remained to be investigated.

**TABLE 1:** Reported biological assays on adzuki extracts.

	Compound/ extract name	Assay type	Reported biology assay	Biological potency	References
1	Adzuki ethyl acetate extract	in vitro	α‐Glycosidase	IC_50_ of 53.74 mg/mL	(Yao et al., 2011)
2	Adzuki beans extract	in vitro	MIC/ MBC (*B. cereus*) MIC/ MBC (*S. aureus*) MIC/ MBC (*E. coli*) MIC/ MBC (*S. typhimurium*)	313/313 μg/mL 313/625 μg/mL inactive	(Gan et al., 2016)
3	Adzuki beans extract	in vitro	TPC TFP FRAP ABTS	1337 mg GAE/100 g DW 941 ± 22.4 mg CE/100 g DW 139 mmol Fe (II)/g DW 95.3 mmol TE/g DW	(Gan et al., 2016)
4	Total phenolics and individual phenolic acids	in vitro	α‐glycosidase DPPH BSA–glucose BSA–MGO	62.57% 88.39 mM TE/g >60% >60%	(Yao et al., 2012)
5	Hot water extract of adzuki beans	in vivo	α‐amylase maltase sucrase‐isomaltase	0.78 mg/mL 2.45 mg/mL 5.37 mg/mL 1.75 mg/mL	(Itoh et al., 2004)
6	Hot water extract of adzuki beans (40% ethanol extract)	in vivo	Lowers postprandial glucose level in streptozocin‐induced diabetic rats		(Itoh et al., 2004)
7	Hot water extract of adzuki beans (40% ethanol extract)	in vivo	Plasma insulin concentration HbA1c, ACR levels postprandial glucose level DPPH	(CV 46.2%) (CV 31.4%) (CV 122.6%) (CV 30.9%) 2.1 μg/mL	(Itoh et al., 2009)
8	Hot water extract of adzuki beans (40% ethanol extract)	in vivo	Liver TC liver TG liver PL serum TC, TG, PL	CV 20.4% CV 5.3% CV 6.9% same as above	(Itoh et al., 2009)
9	Adzuki black (phenolic extracts)	in vivo	α‐glucosidase DPPH FRAP	IC_50_, 26.28 mg/mL 5.70 μmol Trolox equiv/g 34.46 mmol Fe^2+^ equiv/100 g	(Sreerama et al., 2012)
10	Adzuki red (phenolic extracts)	in vitro	Inhibits pancreatic lipase DPPH FRAP	7.32 to 9.85 mg/mL 4.44 μmol Trolox equiv/g 27.39 mmol Fe^2+^ equiv/100 g	(Sreerama et al., 2012)
11	Adzuki beans extract	in‐vitro	Nitric oxide (NO) production inhibition (%)	21.46%	(Jiang et al., 2014)
12	Adzukisaponin I	in vitro	NO production (IC_50_)	>50 μM	(Jiang et al., 2014)
13	Adzukisaponin II	in vitro	NO production (IC_50_)	>50 μM	(Jiang et al., 2014)
14	Adzukisaponin III	in vitro	Nitric oxide production in LPS‐ activated RAW264.7 macrophages was suppressed with IC_50_ values of 13 μM to 24 μM	15 μM	(Jiang et al., 2014)
15	Adzukisaponin IV	in vitro	Suppresses NO production	>50 μM	(Jiang et al., 2014)
16	Adzukisaponin V	in vitro	Proliferation of 3 T3‐L1 preadipocytes in a dose‐dependent manner	14 μM	(Jiang et al., 2014)
17	Angulasaponin A	in vitro	Suppresses NO production	22 μM	(Jiang et al., 2014)
18	Angulasaponin B	in vitro	Suppresses NO production	14 μM	(Jiang et al., 2014)
19	Angulasaponin C	in vitro	Suppresses NO production	13 μM	(Jiang et al., 2014)
20	Angulasaponin D	in vitro	Suppresses NO production	13 μM	(Jiang et al., 2014)
21	Angularin A	in vitro	Suppresses NO production	22 μM	(Jiang et al., 2014)
22	Angularides B	in vitro	Suppresses NO production	> 50 μM	(Jiang et al., 2014)
23	(+)‐catechin 7‐*O*‐*β‐D‐*glucopyranoside (C7G)	in‐vitro	α‐amylase α‐glucosidase	0.74 mg/mL 0.085 mg/mL	(Kuriya et al., 2019)
24	+)‐epicatechin 7‐O‐β‐d‐glucopyranoside (E7G)	in vitro	α‐amylase α‐glucosidase	0.4 mg/mL 0.051 mg/mL	(Kuriya et al., 2019)
25	(90%) polysaccharides from Adzuki beans	in vitro	FBG ↓ TG ↓ hepatic glycogen ↑, insulin resistance↓, HDL‐C ↑	doses 100, 200 and 400 mg/kg BW	(Wu et al., 2020)
26	Adzuki beans water ext.	in vivo	↓lipid accumulation in 3 T3‐L1 cells, bodyweight ↓, adipose tissue weight↓	1 mg/mL	(Lee et al., 2019)
27	Black adzuki beans ext.	in vitro	High glucose‐induced glucotoxicity↓		(Lee et al., 2019)
28	Black adzuki beans ext.	in vivo	HOMA‐IR↓, hyperglycemia ↓		(Han, Fukushima, Shimizu, et al., 2003)
29	Resistant starch of adzuki beans	in vivo	Serum cholesterol ↓ bile acid excretion↑		(Han, Fukushima, Kato, et al., 2003)
30	Adzuki polyphenols	in vivo	Serum cholesterol ↓, LDL‐P ↓		(Nishi et al., 2008)
31	40% ethanol fraction of adzuki beans	in vivo	Serum cholesterol ↓, serum TG ↓		(Itoh et al., 2009)
32	Phenolic ext. of adzuki beans	*clinical*	Serum TG↓ pancreatic lipase (%)	15%–17% reduction 29%	(Maruyama et al., 2008)

**FIGURE 1:**
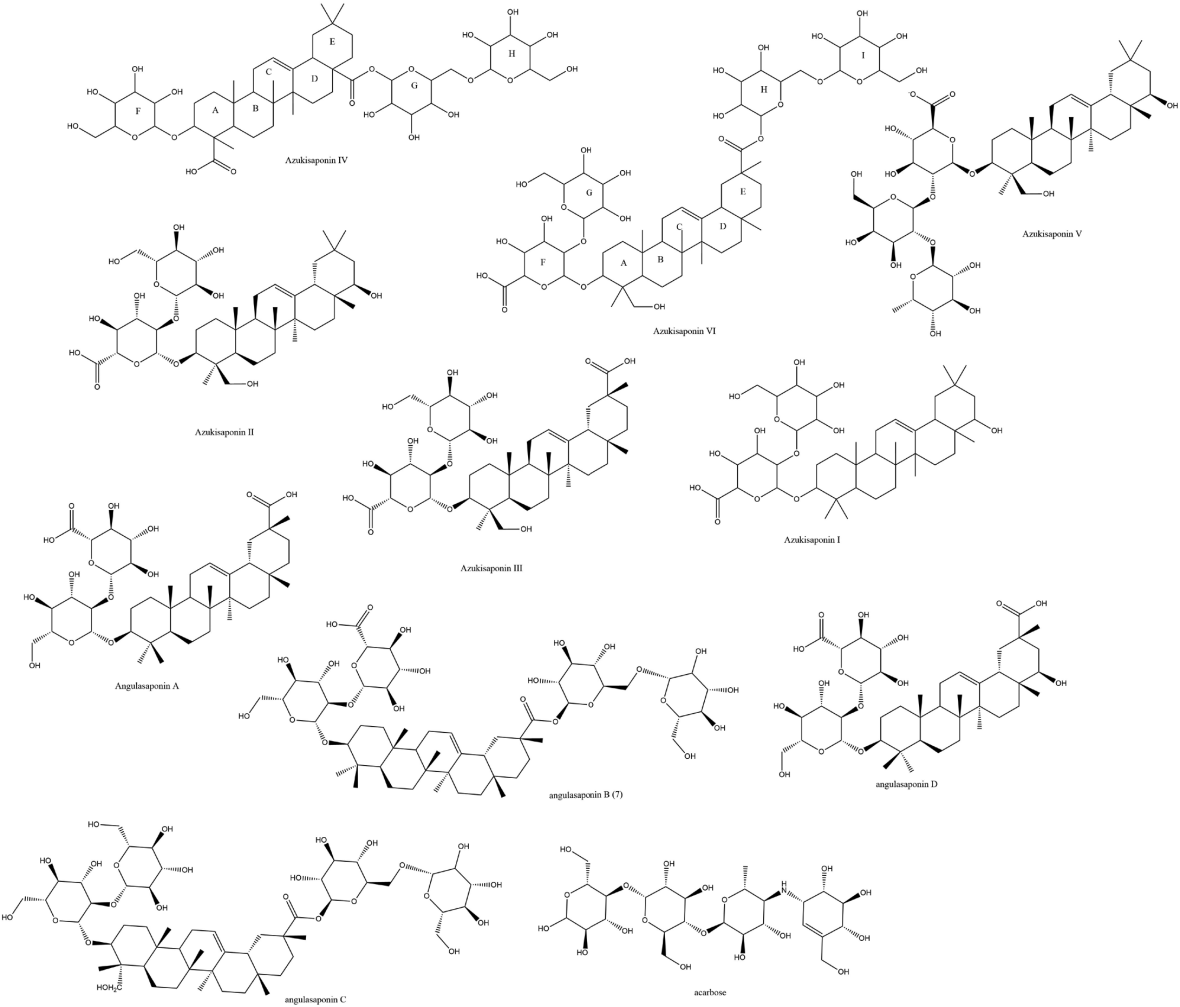
The screened scaffolds of adzuki beans saponin compounds and a positive drug acarbose.

Pure single saponin compounds from adzuki beans are not available until now, and all the data reported the whole activity of the crude saponin extract, which have its known disadvantages (Newman, 2021). Crude extracts are abundant as promiscuous players with multiple bioactivities, and with the advent of Artificial Intelligence (AI) techniques, the use of these claimed bioactive extracts with their main components referred to as the bioactive materials is no more accepted since it misleads large compound databases and other libraries for high‐throughput screening (Rodrigues, 2019). These databases captured a significant number of compounds that will hit screens for several reasons just because they were reported invalidly as biologically active. In silico computational approaches served to overcome this problem and are invaluable nowadays in order to investigate bioactive molecules in their binding sites, their types, and stability of interactions, and in many cases to identify unknown targets (Torky et al., 2021). The cost‐effectiveness and time saving conferred by such techniques encouraged researchers to revive drug discovery from natural products. This grew in line with the limiting ethical laws of animal use, which rationalized the strategy of using in silico investigations for lead identification. Furthermore, false‐negative molecules can be optimized for better binding in receptor sites by medicinal chemists. Diabetes and obesity are correlated strongly to starch metabolic disorders, and starch digestive enzymes were targeted to reduce postprandial sugar and reduce complications that are of major global concern (Al‐Goblan et al., 2014). The mechanism of starch digestion encompasses four major enzymes, pancreatic and salivary‐amylases, maltase‐glucoamylase, and sucrase‐isomaltase that conduct the hydrolysis process from starch to glucose. In this study, four α‐glucosidases were selected, which include human pancreatic α‐amylase, human lysosomal acid‐α‐glucosidase, GAA, the N‐terminal sucrase‐isomaltase, and the C‐terminal subunit of human maltase‐glucoamylase to demarcate the activity of more than 10 adzukisaponin compounds as potential antidiabetic and anti‐obesity agents (Figure 1). While continuing our work on adzuki beans (Liu & Xu, 2016), we embarked on this study aiming to virtually screen the triterpenoidal saponins isolated from adzuki beans against four vital carbohydrate‐digesting enzymes through high‐precision molecular modeling and dynamics mechanisms with Molecular Mechanics energies combined with Gerneralized Born and Surface Area (MMGBSA) Continuum solvation area energy calculations to recognize and predict the molecular basis of the proposed anti‐obesity potential. The proposed hypothesis is that saponins are responsible for the anti‐obesity effects of adzuki beans. All saponins isolated from *V. angularis*, namely, adzukisaponin I, II, III, IV, V, and VI, angulasaponin A, B, C, and D (Iida et al., 1997, 1999) were included. The selection of the study was due to the wide folk use of adzuki beans for weight loss and the structural similarity between acarbose, the standard antidiabetic drug, and many of the adzukisaponins.

## 2 MATERIALS AND METHODS

### 2.1 Protein and ligand preparation

The four selected carbohydrate‐digesting proteins for this study with their co‐crystallized ligands were the human pancreatic α‐amylase in complex with montbretin A (Pdb ID:4w93), human lysosomal acid‐α‐glucosidase, GAA, in complex with acarbose (Pdb ID:5nn8), the N‐terminal sucrase‐isomaltase with kotalanol (Pdb ID:3lpp), and the C‐terminal subunit of human maltase‐glucoamylase in complex with acarbose (Pdb ID:3top) whose crystallographic data were obtained from the Protein Data Bank (PDB) website (https://www.rcsb.org/).

The Schrödinger platform was employed to prepare the chosen proteins via Protein Preparation tool in order to add hydrogen and correct problems like incomplete loops or side chains, flipped residues or unclear protonation states. Forcefield of OPLS2005 was applied for energy minimization after optimization of the preprocessed protein; moreover, ionization states were selected as 7.4 pH and water molecules were kept based on their important role in interactions (AbdelRazek et al., 2023; Torky et al., 2021).

### 2.2 Receptor grid generation and ligand preparation

**FIGURE 2:**
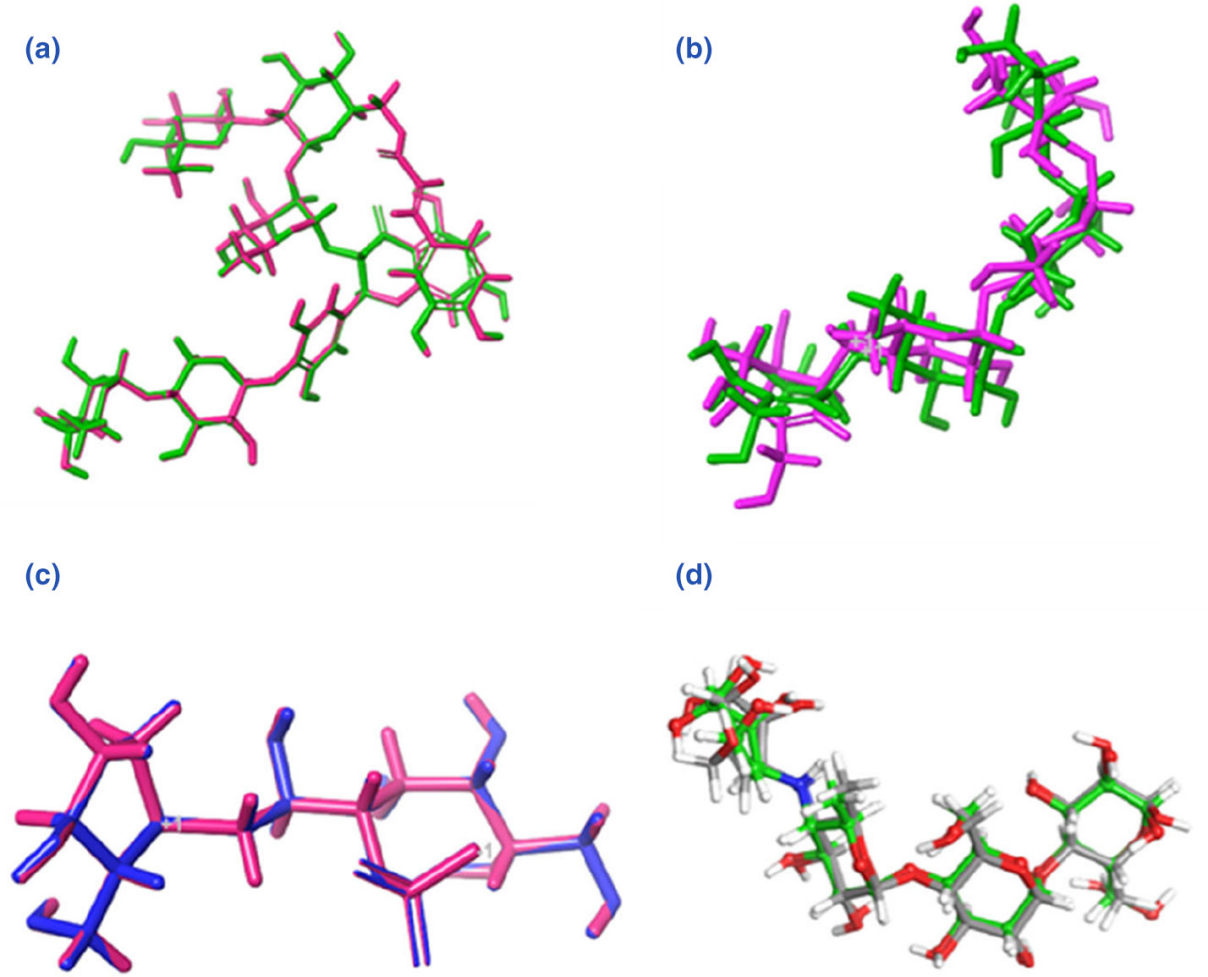
The validation RMSD values in the four selected enzymes, (a) 4w93, (b) 3top, (c) 3lpp, and (d) 5nn8.

The native ligands like montbretin (4w93), kotalanol (3lpp), and acarbose (5nn8 and 3top) were picked and defined to locate the docking position for the wizard using the Receptor Grid Preparation in Maestro 11.6. Furthermore, the native ligands’ structures were copied to separate files. Their structures were subjected to ligprep tool for energy minimization in three‐dimensional (3D) low‐energy formats up to 2500 iterations, and redocked using the same ligand docking protocol of the chosen compounds library in each protein to calculate the root‐mean‐square deviation (RMSD) and validate the procedures (Figure 2).

### 2.3 Molecular docking

The Glide ligand docking protocol in Maestro was utilized to evaluate the binding affinity and stability of each of the selected compounds in the enzymatic groove of the four prepared proteins through high extra precision standards (XP). The filtering results are used to generate the complex Glide XP scores, which are created by ranking single ligand poses of separate chemical entities to separate those that bind strongly from compounds that bind weakly or are inactive through Glide score and using the “XP Pose rank” in case of generating conformers of the same ligand and considering the following equation that involves both E‐model and Glidescore values.

$$\text{Docking score}\;\left(\text{Glide score}\right)=a\times \mathrm{vdW}+b\times \text{Coul}+\text{Hbond}+\text{Lipo}+\text{Metal}+\text{RotB}+\text{site}.$$

The E‐model score expresses more the protein–ligand coulomb–VdW (van der Waals) energy considering also the Glide score. The physical aspects of the binding affinities, lipophilicity, hydrogen bonds, and protein–ligand coulomb–VdW energies are represented by the Glidescore term. The grid box dimensions were specified in the grid generation tool as 14 Å × 14 Å × 14 Å, which allows the selected ligands’ fitting; moreover, van der Waals factor was set to 1.00 within an OPLS2005 forcefield and workload control factor of 0.25. Finally, the poses were selected based on the lowest GlideXP score and E‐model provided by poses that bind vital core amino acids in the enzymatic grooves. All protein native ligands were redocked in the primary X‐ray structures of the proteins to measure their RMSD.

### 2.4 MD simulation

The docking results of targeted proteins were analyzed and one compound from each study was selected for protein–ligand stability analysis at 100 ns molecular dynamics (MD) simulation using Visual Molecular Dynamics (VMD) (William, 1996) and Nanoscale Molecular Dynamics (NAMD) (Phillips et al., 2020) tools. The input files required for simulation were generated in AmberTools 21 (Case et al., 2021), therein the Antechamber was used to generate the ligand topology files while the missing hydrogens were added by using LeaP program (Case et al., 2005). Each ligand–protein complex was then solvated in a solvation box of 10 Å containing TIP3P water model (Duan et al., 2003; Phillips et al., 2020). To further neutralize the systems, counter Na^+^ and Cl^−^ ions were added to the complexes. To remove clashes, the complexes were minimized by using FF14SB and General Amber Force Fields (GAFFs) for protein and ligand, respectively (Duan et al., 2003). Finally, the solvation systems were subjected to three additional equilibrations at 200, 250, and 300 K. The processed ligand–protein complexes were then subjected to 50 ns simulation, and the MD trajectories were saved at every 2 ps interval. The trajectories were analyzed by CPPTRAJ (Weinzierl, 2021) and R package (Grant et al., 2021).

## 3 RESULTS

### 3.1 Docking interaction

Detailed molecular, ionic, hydrophobic, and van der Waals docking interactions, are listed in Tables 2, 3, 4, 5 for the four enzymes, human pancreatic α‐amylase, human lysosomal acid‐α‐glucosidase, the N‐terminal sucrase‐isomaltase, and the C‐terminal subunit of human maltase‐glucoamylase.

**TABLE 2:** Different interactions of the selected compounds in α‐amylase protein.

	Compound name	XP‐Gscore	Hydrogen bonds	Number of H bonds	Hydrophobic interactions	Salt bridge
1	Adzukisaponin I	−9.393	Ile235(1.83), Lys200(1.97), Glu240(1.91,1.84)	4	Ile235, Leu 237, Tyr151	
2	Adzukisaponin II	−9.66	Glu240(1.76)	1	Ala307, Leu162, Trp59, Ile235, Tyr151	
3	Adzukisaponin III	−7.412	Asp300(2.09), Asp197(2.75)	2	Tyr62, Trp58, Trp59, Ile148, Leu165, Leu162, Ile235	
4	Adzukisaponin IV	−13.078	Lys261(2.08), Thr264(2.41), Gly308(1.88), His201(2.19), Glu240(1.65)	5	Ala260, Leu162, Tyr151, Leu237, Ile235, Ala307, Gly306, Gly309	
5	Adzukisaponin V	−8.833	Thr163(2.33)	1	Tyr62, Trp59, Leu162, Leu165, Ala106	
6	Adzukisaponin VI	−15.599	Glu240(2.03, 1.92), Asp353(2.69), Asp356(1.70), Asp300 (1.70)	5	Trp357, Val354, Tyr151, Leu162, Ile235, Gly306, Thr163, Leu237, Trp58, Trp59	Lys200(2.61)
7	Angulasaponin A	−7.913	Ala310(1.89), Tyr151(2.30), Lys200(1.59), Glu240(1.85)	4	Trp269, Ala310, Tyr151, Ile235, Leu237	Lys200(4.37)
8	Angulasaponin B	−14.621	Lys261(1.94), Gly308(2.37), Thr264(1.93), Glu233(2.62), Asp197(1.64)	5	Tyr151, Leu162, Ile235, Gly307, Trp58, Trp59, Tyr62, Ala198, Val354, Trp357, Trp269, Ala307, Leu237, Leu165	Lys261(3.02)
9	Angulasaponin C	−13.759	Lys261(2.57), Thr264(2.17), Ser311(2.32), Ala310(2.23), Gly308(2.63), Asp300(1.88)	6	Leu162, Leu237, Tyr151, Trp357, Val354, Ala260, Trp269, Trp58, Trp59	
10	Angulasaponin D	−9.56	His201(2.08), Lys200(1.95), Asn352(1.92)	3	Tyr151, Leu162, Ile235	
11	Acarbose	−13.082	Thr163(2.33)	5	Ile235, Leu162, Leu165, Trp58, Trp59, Tyr62, Val98, Ala198, Tyr151	
12	Soraphen	−5.433	Tyr62(π‐π stacking 5.01), Trp58(π‐π stacking 5.05)		Trp58, Trp59, Tyr62, Leu162, Trp357, Ile235	
13	native_3L9	−22.034	Glu233(1.81), Glu240(1.89,1.94), Asp197(1.71,1.78), Arg195(2.00), Tyr62 (π ‐π stacking 5.37), His290(π‐π stacking5.37)	6	Trp58, Trp59, Tyr62, Leu162, Ala198, Leu165, Val98, Tyr151, Leu237, Ile235	

**TABLE 3:** Different interactions of the selected compounds in α‐glucosidase protein.

	Compound name	XP‐Gscore	Hydrogen bonds	Number of H bonds	Hydrophobic interactions	Salt bridge
1	Adzukisaponin I	−7.501	Arg600(2.34), Asp616(1.82,1.87), Asp518(1.94), Asp404(1.83), Asp282(1.76)	6	Phe525, Trp481, Leu405, Met519, Trp376, Trp516, Ile441, Trp613, Tyr292, Trp618, Phe649, Leu650, Leu677, Leu678	
2	Adzukisaponin II	−6.029	Asp282 (2.12, 2.21, 2.04)	3	Phe525, Trp481, Met519, Trp376, Trp516, Trp613, Tyr292, Trp618, Phe649, Leu650, Leu283, Ala284, Leu677, Leu678	
3	Adzukisaponin III	−5.901	Asp616 (1.94)	1	Phe525, Trp481, Trp376, Tyr292, Trp618, Phe649, Leu650, Leu283, Ala284, Leu677, Leu678	
4	Adzukisaponin IV	−11.585	Trp481(1.86,1.96), Asp404(2.48), Arg600(2.48), Asp616(1.68), Asp282(1.69)	6	Phe525, Trp481, Trp376, Trp618, Phe649, Leu650, Leu677, Leu678, Pro482, Leu405, Ile441, Trp516, Trp613, Met519	Arg411(4.82)
5	Adzukisaponin V	−7.935	Asp616(2.15), Arg600(2.00), Ser676(1.84)	3	Phe525, Trp481, Trp376, Trp618, Phe649, Leu650, Leu677, Leu678, Met519, Tyr292, Ala284	
6	Adzukisaponin VI	−11.173	Val480(2.17), Asp404(2.70,1.88), His674(2.62), Asp616(1.67), Arg600(2.02), Asp282(1.68)	7	Phe525, Trp481, Trp376, Phe649, Leu650, Leu677, Leu678, Met519, Tyr292, Ala284, Pro482, Val480, Leu405, Ile526, Trp516, Trp613, Leu283	Lys470(4.93)
7	Angulasaponin A	−7	Arg281(2.31), Asp282(2.00,2.19), Ala284(1.87), Asp616(2.76)	5	Phe525, Met519, Ala284, Ile526, Leu283, Ala555	Lys479(2.89)
8	Angulasaponin B	−10.285	Lys479(1.66), Asp616(1.96,1.71,1.71), Arg600(1.88), Asp518(1.83)	6	Phe525, Trp481, Trp376, Phe649, Leu650, Leu677, Leu678, Met519, Tyr292, Ala284, Pro482, Val480, Leu405, Ile526, Trp516, Trp613, Ile441	Lys479(4.19)
9	Angulasaponin C	−11.335	Pro482(1.87,1.97), Arg600(2.18), Asp616(1.66,1.71), Asp518(1.91), Ser410(2.15)	7	Phe525, Trp481, Trp376, Phe649, Leu650, Leu677, Leu678, Met519, Tyr292, Pro482, Val480, Leu405, Ile526, Trp516, Trp613, Ile441	
10	Angulasaponin D	−6.353	Asp282(1.68, 1.80), Asp518(2.58), Asp404(2.27)	4	Phe525, Trp481, Trp376, Phe649, Leu650, Leu677, Leu678, Met519, Trp516, Trp613, Ile441	
11	Acarbose	−9.766	Asp282(1.93, 2.33, 2.11), Phe525(2.11), Asp616(2.23,1.71), Asp404(1.95)	7	Phe525, Trp481, Trp376, Phe649, Leu650, Met519, Trp516, Trp613, Ile441, Ile526, Ala555, Leu405, Tyr292	
12	Soraphen	−4.572			Phe525, Trp481, Trp376, Phe649, Leu650, Trp516, Trp613, Ile441, Ala284, Leu405	
13	Thiazolideneine	−4.237	Ser676(1.72), Gly651(2.09)	2	Leu677, Leu678, Leu650	

**TABLE 4:** Different interactions of the selected compounds in the maltase protein.

	Compound name	XP‐Gscore	Hydrogen bonds	Number of H bonds	Hydrophobic interactions	Salt bridge
1	Adzukisaponin I	−5.844	Asp231 (2.14), Lys509(1.89), Trp435(1.80)	3	Val434, Phe604, Pro436, Leu233, Trp327, Met473, Phe479, Trp435	
2	Adzukisaponin II	−4.274	Glu233(2.49)	1	Ile480, Phe479, Val434, Trp435, Trp327	Lys509(2.80)
3	Adzukisaponin III	−5.757	Asp503(1.93,2.01), Lys509(1.97), Asp236(1.86)	4	Val605, Leu502 Trp327, Phe479, Tyr634, Trp581	
4	Adzukisaponin IV	−9.165	Lys509(2.24), Asp231(1.88), Asp571(2.17), Glu433(1.84)	4	Val605, Leu502 Trp327, Phe479, Tyr634	
5	Adzukisaponin V	−6.209	Phe479(2.39, 1.92), Glu433(2.56), Arg230(1.98), Gln232(2.01), Lys509(2.63), Asp503(2.43)	7	Val434, Phe604, Pro436, Leu233, Trp327, Met473, Phe479, Trp435	
6	Adzukisaponin VI	−11.313	Lys509(1.90), Asn237(2.18), Leu233(1.93), Pro234(1.93), Asp231(1.97), Asp571(2.19)	6	Val605, Leu502, Phe604, Pro234, Leu233, Trp327, Met473, Phe479, Trp435	
7	Angulasaponin A	−5.723	Lys509(2.60), Asp231(2.50)	2	Ile480, Phe479, Trp435, Trp327, Met473, Leu233, Phe604	
8	Angulasaponin B	−9.792	Asp571(2.25), Asp231(2.11), Lys509(2.34), Asp632(1.81), Trp435(2.02), Val434(2.19)	6	Leu502, Val434 Phe604, Pro436, Leu233, Trp327, Met473, Phe479, Trp435, Ile480	Lys362(2.70)
9	Angulasaponin C	−11.751	Leu233(2.14), Asn239(2.27), Asp571(1.77), Asp231(2.21), Gln232(2.51)	5	Val605, Leu502, Phe604, Pro234, Leu233, Trp327, Met473, Phe479, Trp435	
10	Angulasaponin D	−7.266	Asp231(2.01), Lys509(2.59)	2	Phe604, Leu233, Trp327, Met473, Phe479, Ile480	
11	Acarbose	−16.048	Lys509(1.89,2.21), Asp231(1.83,2.57), Asp571(2.79, 1.68, 1.75), Asp355(2.11, 1.98, 2.43)	9	Leu502, Phe604, Leu233, Trp327, Met473, Phe479, Trp435, Trp470, Ile392, Ile356, Trp568	Asp571(2.76)
12	Soraphen	−3.146			Ile480, Phe479, Trp435, Trp327, Met473, Leu233, Phe604	Trp435(5.33) π‐π stacking
13	Kotalanol	−12.559	Asp231(1.82), Lys509(1.89), Asp571(1.77, 2.80), Trp327(5.87, π‐cation), Asp355(1.81), Asp472(4.80), His629(2.60)	8	Phe479, Leu233, Tyr242, Trp327, Trp568, Phe604, Ile356, Trp470, Ile392, Met473, Trp435	Asp472 (3.80, salt bridge), Asp571(4.55, salt bridge)

**TABLE 5:** Different interactions of the selected compounds in the sucrase protein.

	Compound name	XP‐Gscore	Hydrogen bonds	Number of H bonds	Hydrophobic interactions	Salt bridge
1	Adzukisaponin I	−7.765	Asp1157(1.74), Lys1164(1.91), Gln1158(1.83), Lys1460(2.39)	4	Phe1560, Phe1427, Trp1355, Trp1369, Pro1159, Met1421, Tyr1167, Tyr1251	
2	Adzukisaponin II	−8.057	Gly1365(1.84,2.08), Lys1460(2.11), Gly1588(2.66), Gln1561(2.03)	5	Phe1560, Phe1427, Trp1355, Trp1369, Phe1559, Pro1159, Met1421, Ile1587	
3	Adzukisaponin III	−6.01	Arg1156(2.34), Arg1455(1.97), Asp1454(1.82), Ser1452(2.45), Glu1451(2.79,1.54,2.22)	7	Phe1560, Phe1427, Trp1355, Trp1369, Phe1559, Pro1159, Met1421, Tyr1251	Arg1510(4.60), Arg1455(4.81)
4	Adzukisaponin IV	−12.518	Lys1164(1.57), Tyr1162(2.43), Gln1158(2.37), Asp1157(2.01), Asp1279(1.66,1.93), His1584(2.09), Asp1526(1.80,1.88), Glu1138(2.13), Asp1532(1.91,1.74), Gln1533(2.04)	13	Tyr1167, Ala1530, Tyr1162, Pro1160, Trp1523, Tyr1251, Ile1280, Ile1315, Trp1418, Trp1355, Met1421, Phe1560, Phe1559, Trp1369, Pro1159, Ile1587	Lys1164 (3.95)
5	Adzukisaponin V	−8.098	Asp1157(2.15,2.68), Gln1158(1.74, 2.38), Glu1451(1.80), Lys1460(2.43)	6	Phe1560, Phe1427, Trp1355, Trp1369, Pro1159	
6	Adzukisaponin VI	−12.551	Lys1164(1.71), Pro1159(2.62), Gln1158(2.08), Asp1157(1.66,1.59), Asp1526(1.94), His1584(2.12), Asp1279(1.82,1.67), Asp1532(1.71), Gln1533(2.02)	11	Tyr1167, Ala1530, Tyr1162, Pro1160, Trp1523, Tyr1251, Ile1280, Ile1315, Trp1418, Trp1355, Met1421, Phe1560, Phe1559, Trp1369, Pro1159, Phe1427	Lys1164(3.06)
7	Angulasaponin A	−7.605	Tyr1251(2.45), Arg1156(1.94), Glu1451(1.66,1.86), Ser1452(1.94,1.69, Asp1370(1.80)	7	Phe1559, Phe1560, Ile1587, Tyr1251, Trp1355, Pro1159, Trp1369	
8	Angulasaponin B	−10.723	Lys1536(1.75), Asp1157(2.13), Lys1164(2.02), Asp1279(1.88), Asp1420(2.38), Asp1526(1.93,1.89), Asp1532(1.90,1.70)	9	Tyr1167, Ala1530, Tyr1162, Pro1160, Trp1523, Tyr1251, Ile1280, Ile1315, Trp1418, Trp1355, Met1421, Phe1560, Phe1559, Trp1369, Pro1159, Phe1427	Lys1536(4.61)
9	Angulasaponin C	−13.103	Asp1532(1.58,2.26), Gln1533(2.07), Glu1138(1.79), Lys1164(2.30), Tyr1162(2.12), Trp1369(2.24), Asp1526(1.91,1.68), Asp1279(2.09), His1584(2.14)	11	Tyr1167, Ala1530, Tyr1162, Pro1160, Trp1523, Tyr1251, Ile1280, Ile1315, Trp1418, Trp1355, Met1421, Ile1587, Phe1560, Phe1559, Trp1369, Pro1159	
10	Angulasaponin D	−8.406	Trp1369(2.12), Lys1364(2.02), Glu1451(1.70), Arg1156(2.32), Gln1158(1.98)	5	Trp1369, Trp1355, Phe1560, Ile1587, Pro1159, Leu1367, Tyr1251	Arg1377(4.29)
11	Acarbose	−12.303	Arg1510(1.82), Arg1156(2.32), Asp1157(2.46), Lys1460(1.99), Asp1526(2.02, 1.77, 2.01), His1584(2.14), Asp1279(1.83)	9	Tyr1167, Trp1523, Tyr1251, Ile1280, Ile1315, Trp1418, Trp1355, Met1421, Ile1587, Phe1560, Phe1559, Trp1369, Pro1159	
13	Thiazolideneine	−5.219	Asp1279(1.91), His1584(1.74)	2	Phe1559, Trp1523, Ile1315, Ile1280, Trp1355, Met1421, Tyr1251, Trp1418	

### 3.2 MD simulation

The MD trajectories were analyzed to calculate the RMSD, root mean square fluctuation (RMSF), and radius of gyration (Rg) of all selected ligand–protein complexes, separately. The RMSD plot of sucrase–angulasaponin C complex showed that the systems attained equilibrium at 2 ns with an RMSD value of 1 Å (Figure S1a). After equilibration, the complex did not show any major deviation in the RMSD value till the end of simulation, which indicated that the protein did not undergo major conformational changes during simulation. The maximum value of RMSD attained by the protein was 1.5 Å at 80 ns. The Rg described the compactness of the protein structure where higher values inferred that the protein faced some unfolding events during simulation. The Rg plot of sucrase–angulasaponin C complex showed that the system had a stable Rg value starting at 28.2 Å and reached 28.4 Å at equilibration. It gradually increased to 28.6 Å at 70 ns before attaining the previous values toward the end of simulation. The overall results showed that the complex did not face any unfolding event during simulation and remained compact (Figure S1b). Similarly, the RMSF analysis revealed that no major fluctuations were observed in protein residues. The higher RMSF value showed the loop regions while the lower RMSF indicated the rigid secondary structures. The protein residues did not show major fluctuations, except for the N‐terminals. Meanwhile, other residues remained rigid indicating the stable secondary structures (Figures S1c and S2).

The complex of maltase with angulasaponin VI was selected for the MD simulation. The RMSD plot of maltase–angulasaponin VI complex backbone atoms showed that the RMSD values of the complex attained equilibrium at 5 ns where the RMSD value reached 2 Å before it was gradually increased to 2.5 Å at 40 ns. After 40 ns, it gradually decreased to 2 Å at 60 ns and deviated in the range of 2–2.25 Å till the end of simulation (Figure S3a). The Rg plots showed that the protein bound to the ligand remained compact, as the Rg values did not show major variations and remained between 28.7 and 28.9 Å during whole simulation time, after being equilibrated at 5 ns (Figure S3b). This indicated that the ligand did not induce any unfolding event in the protein structure. Similarly, the RMSF plots show that the major fluctuations were observed in the residues from 400 to 410 (Figure S4) and C‐terminal restudies (Figure S3c).

In case of the glucosidase enzyme, the complex with adzukisaponin IV was selected for MD simulation analysis. The RMSD of the backbone atoms of the glucosidase complex did not show major deviations as the plot showed that the RMSD gradually increased to 1.5 Å at 15 ns and then attained stability in the range of 1.5–2 Å till the end of simulation (Figure S5a). The Rg plot showed deviations of 0.6 Å till 20 ns and then attained stability in the range of 28.3–28.5 Å till the end of simulation (Figure S5b). Similarly, the RMSF analysis showed no major fluctuation in the glucosidase residues, except for the loop regions. Additionally, some minor fluctuations were observed, and the overall structure did not show flexibility (Figure S5c). The stable amino acid contacts are shown in Figure S6.

For the α‐amylase protein, angulasaponin B and adzukisaponin VI‐complexes were selected for stability analysis. The RMSD of α‐amylase–adzukisaponin VI complex attained stability in the range of 1.25–1.5 Å after 10 ns and then remained in the same range till the end of simulation. While, α‐amylase–angulasaponin B complex remained less than 2 Å till 20 ns and then showed deviations for some time and then attained stability in the range of 2–2.5 Å till the end of simulation with some minor deviations at 60 and 75 ns (Figure S7a). A similar trend was observed in Rg analysis as the Rg of adzukisaponin VI remained in the range of 23–23.2 Å throughout the simulation, while the Rg of angulasaponin B showed stability till 20 ns at 23–23.3 Å and then gradually increased to 23.75 Å at 60 ns. It again attained stability toward the end of simulation in the range of 23.3 Å (Figure S7b). The RMSF analysis showed that the loop regions in the angulasaponin B complex exhibited more fluctuations than the adzukisaponin VI complex as the RMSF values increased to 6 Å at the residues ranging from 140 to 160 in the angulasaponin B complex (Figure S7c). The remaining residues remained rigid with minor fluctuations (Figure S8).

### 3.3 MMGBSA

Molecular mechanics Generalized Born Surface Area (MM/GBSA) method was used to calculate the total binding free energy (ΔGtotal) for both complexes. The ΔGtotal value is usually used to estimate the stability of protein–ligand complex. The lower values of ΔGtotal indicate that the complex is more stable and vice versa. It was computed as a sum of the protein–ligand complex and the difference of protein and its ligands’ free energies. The total binding free energy estimated using MM/GBSA model is the outcome of the contribution of various protein–ligand interactions, such as van der Waals energy (ΔEvdW), electrostatic energy (ΔEele), and ΔGGB (electrostatic contribution to solvation free energy by Generalized Born (GB)). The total binding free energies are given in Table 6. The binding energy contributions of the α‐amylase complexes are compared and shown in Figure S9. The ΔEvdW contribution of α‐amylase–angulasaponin B complex was more than that of α‐amylase–adzukisaponin VI complex, but the electrostatic contribution of amylase–adzukisaponin VI complex was even more. The GB contribution showed that α‐amylase–adzukisaponin VI complex has a higher GB value than α‐amylase–angulasaponin B complex. The total binding free energies of both complexes were 46.77 for α‐amylase–adzukisaponin VI complex and −74.18 for α‐amylase–angulasaponin B complex (Figure S9).

**TABLE 6:** The binding free energy contributions of the complexes.

Energy components	Sucrase–Angulasaponin		Maltase–Adzukisaponin VI		Glucosidase–Adzukisaponin IV		α‐Amylase–Adzukisaponin VI		α‐Amylase–Angulasaponin B	
	Start	End	Start	End	Start	End	Start	End	Start	End
ΔEvdW	−53.47	−58.27	−80.34	−79.27	−29.08	−53.35	−65.14	−88.42	−71.79	−99.31
ΔEele	−27.84	−41.57	126.26	141.93	172.61	173.00	−41.37	14.29	−48.58	11.13
ΔEGB	53.98	61.56	−100.71	−119.20	−161.41	−147.16	68.12	21.54	64.93	25.56
ΔEsurf	−7.16	−7.55	−11.44	−11.23	−3.80	−6.09	−8.38	−10.38	−8.68	−11.56
ΔGgas	−81.32	−99.84	45.92	62.66	143.52	119.64	−106.51	−74.12	−120.37	−88.18
ΔGsolv	46.81	54.01	−112.15	−130.43	−165.22	−153.25	59.73	11.16	56.24	14.00
ΔGtotal	−34.50	−45.83	−66.23	−67.77	−21.70	−33.60	−46.77	−62.96	−64.12	−74.18

## 4 DISCUSSION

Carbohydrate intake was highly correlated to the metabolic syndrome and its bad prognosis (Hyde et al., 2019). Dietary intake with low carbohydrate and high fat proved to be beneficial to metabolic syndrome patients than high carbohydrate and low‐fat one.

Saponins were reported to improve insulin resistance and suppress visceral fat accumulation; for example, in the staple food of *Chenopodium quinoa* Willd. (Li et al., 2022); moreover, sea cucumber extracts revealed favorable effect in obese high‐fat fed rats through its effect on pancreatic lipase, reducing total cholesterol, LDL, and total triglycerides (Guo et al., 2016).

Adzuki beans (*V. angularis)* belonging to the Fabaceae family recorded many phytochemical components, such as pigments, terpenes, phenolics, phytosterols, sterylglycosides (Kojima et al., 1989; Luo et al., 2016), proanthocyanidins (Kawahara et al., 2019; Kawakami et al., 2018), flavonoids, and saponins (Jiang et al., 2014). They are legumes with significant economic importance and are considered dietary staple in many cultures. A growing number of studies revealed their role as anticancer, anti‐obesity, and antidiabetic agents (Kris‐Etherton et al., 2002; Sreerama et al., 2012). A human subject's study reported the marked increase of high‐density lipoprotein (HDL) in volunteers who received the adzuki bean extract with no notable side effects during a period of 8 weeks (Kitano‐Okada et al., 2012; Wang et al., 2022). For diabetic noninsulin‐dependent patients, adzuki beans controlled the postprandial glucose level and regulated the oxidative damage induced by inflammation in ailments like cancer, CVD, and atherosclerosis via their phenolic components (Luo et al., 2016). Upon studying the black adzuki bean extract and its effect on pancreatic β‐cells in high glucose‐induced glucotoxicity condition, cells’ viability was ameliorated and restored to the normal glucose level situation; furthermore, insulin secretion was controlled to reduce the insulin resistance index in high‐fat diet‐induced glucose‐intolerant obese C57BL/6J mice (Kim et al., 2016).

Additionally, black adzuki beans affected the adipocytes in a positive anti‐adipogenic way where it raised gene expression of lipolytic genes as adipose triglyceride lipase (ATGL) and hormone‐sensitive lipase (HSL) and lowered the messenger RNA (mRNA) expression of transcription factors like peroxisome proliferator‐activated receptor gamma (PPARγ) and CCAAT/enhancer‐binding protein α (C/EBPα) as well as cell proliferation, related to adipogenesis (Kim et al., 2015). A detailed description of the reported obesity‐related research of adzuki beans is shown in Table 1.

### 4.1 Sucrase‐isomaltase

Sucrase‐isomaltase (SI) is another carbohydrate‐digesting enzyme belonging to the GH31 subfamily for hydrolyzing the terminal starch products, in the form of 1,4‐and/or 1,6‐linked oligosaccharides with overlapping specifications. This binding site was extensively analyzed for the binding of 10 adzukisaponins together with acarbose and kotanalol as control active molecules. An overview of the SI active site revealed three characteristic features, the buried hydrophilic subsite −1 interacting with the nonreducing part of the substrate, the shallow hydrophilic surface subsite +1 interacting with the reducing terminals, and another hydrophobic +1 subsite also involved in nonspecific contacts with the reducing groups of substrates (Sim et al., 2010). The catalytic reaction takes place between these two hydrophilic subsites to cleave the glycosidic link via Koshland mechanism without altering the anomeric carbon configuration. The conserved Trp327 in most of the GH31 family was important for the 1,6‐linkage specificity. Acarbose‐binding mode was largely influenced by its hydrophilic nature allowing it to score −16.048 Kcal/mol through more than eight ionic hydrogen bond interactions with residues as Lys509, Asp231, Asp571, and Asp355; furthermore, the quaternary nitrogen linker extended a salt bridge to Asp571 seemingly imparting more stability to acarbose fitting in the binding site (Table 5). While most of the tested saponin compounds interacted with Asp231 and Lys509 except adzukisaponin II, only the highest scoring ones, adzukisaponin VI and angulasaponin C, were able to exceed other ligands by a high margin score clearly based on their auspicious three‐dimensional (3D) orientation in the binding pocket exposing them to a larger surface area of hydrophobic contacts in the shallow +1 subsite (Figure S10). This was in accordance with Sim et al. who attributed substrate discrimination to the +1 subsite. By inspecting the two‐dimensional (2D) interactions, Asp571 added more stability and better fitting to the highest two scoring compounds as well as angulasaponin B and adzukisaponin IV. A careful analysis of compounds scoring below −7 Kcal/mol demonstrated that they were incapable of interacting with the vital amino acid residues, which also showed their lack of diglucoside moiety in the triterpenoidal E ring.

Kotanalol‐binding Glide XP score was −12.559 Kcal/mol which was rationalized mainly through the interaction of its hydroxyl groups with the −1 subsite amino acids such as Asp 472, Asp355, Asp571, and His629 as well as salt bridges to Asp571 and Asp472. Kotanalol showed much tighter binding to the SI than acarbose and this was largely due to more hydrogen bonds formed in the −1 subsite, interactions with the hydrophobic patches as Phe479, Leu233, Tyr242, Trp327, Trp568, Phe604, Ile356, Trp470, Ile392, Met473, and Trp435. Kotanalol results and interactions were slightly better than angulasaponin C, probably due to the formation of about nine ionic hydrogen bonds encompassing two salt bridges with Asp472 and Asp571 and π‐cation interaction with Trp327. In contrast to the human MGAM (nt MGAM) with its directed preference to the short‐chain 1,4‐oligosaccharides, the N‐terminal of SI (Pdb ID: 3lpp) favored 1,6‐linkage diglucosides, despite their 3‐bond attachment and notable flexibility. Adzukisaponin VI bound to the hydrophobic +1 subsite in such a way as to constrain its 1,6‐linked oligosaccharides through the −1 narrow groove and the hydrophobic adjacent interactions. 1,6‐linked malto‐oligosaccharides fitted in the −1 subsite and 1,2‐linked and /or 1,4‐linked oligosaccharides fitted in the +1 subsite. Angulasaponin C 1, 2 linkages fitted in the +1 subsite and the 1,6‐ linkage fitted in the −1 subsite. Low scoring compounds lacked the 1,6‐linked nonreducing dimaltose. Failure to meet these protein features dramatically declined the GlideXscore and reduced fitting, thus the prospected bioactivity as realized in adzukisaponin I, II, and III and angulasaponin A. Only the 1,6‐linked diglucoside was able to attain the best orientation in the active site (Sim et al., 2010).

The whole simulation analysis was examined and revealed that angulasaponin C interacted stably with Leu233 and Asn237 during the 100 ns simulation time, which indicated the reliability of the previous docking work; moreover, the impact of hydrophobic contacts was evident, since many of them were constant in the simulation run, such as Phe604, Leu233, Trp327, Met473, and Trp435 (Figure S1).

### 4.2 Human maltase‐glucoamylase

Human maltase‐glucoamylase (MGAM‐C) C‐termini is one of the GH31 enzyme subfamily whose members are promising targets for obesity and type 2 diabetes. Previous investigations of the enzyme revealed the existence of additional 21 amino acids conferring two unique subsites, not identified in other GH31 members before, the +2 and + 3 subsites in the MGAM‐C catalytic site turned out to prefer longer substrates (Ren et al., 2011). MGAM‐C binding pocket was illustrated as four subsites as manifested from the standard acarbose binding. Acarbose showed favorable interactions through nine ionic interactions with amino acids Arg1510, Arg1156, Asp1157, Lys1460, Asp1526, His1584, and Asp1279 within a distance between (1.77 and 2.46). Similarly, it shared the same polar residue contacts as with the highest scoring compound angulasaponin C, which are Lys1460, Asp1526. Asp1526, and His1584 (Figure S11; Table 6).

Acarbose extended in the MGAM‐C groove in such a way as to interact with vital residues through polar and hydrophobic contacts in each of the four critical subsites from −1 to +3. The imino nitrogen located between −1 and +1 subsites in the catalytic region. His1584 and Asp1279 formed hydrogen bonds with C3‐OH and C4‐OH of the acarbose cyclitol ring, and several hydrophobic contacts assisted its stabilization as Trp1523, Tyr1251, and Trp1418. In the +1 subsite, the dideoxy amino sugar ring interacted with Asp1157 and with Arg1510 nitrogen atom to form three hydrogen bonds. While Asp1526 interacted with the imino nitrogen of acarbose, π‐π stacking was detected between Phe 1559and Trp1355 and acarviosine rings. In the +2 subsite, Trp1369 stacked with the third ring in acarbose and in the +3 subsite, nonpolar interactions rendered the fourth ring of acarbose more stable through Pro1159 and Phe1560 (Figure S12; Table 5). The −1‐subsite groove is constrained by the hydrophobic patch Trp327, Tyr1251, Phe1559, and Phe1560, which render the 1,6‐glucoside substrates more auspicious as in angulasaponin C, adzukisaponin IV and VI, the highest scoring compounds in our selected molecular skeleton. In the angulasaponin C, the triterpenoidal moiety acted as an effective linker with adequate length to ensure binding of the 1,6‐diglucoside hydroxyl groups to Lys1164, Tyr1162, Glu1138, Gln1533, and Asp1532 and the 1,2‐linked di‐maltose hydroxy‐ and methyl hydroxy moieties to Asp1526, His1584, Asp1279, and Trp1369 (Figure S12). It is worth mentioning that Trp1369 served to constrain the flexibility of the 1,6‐linked sugars to guide the catalytic process. MGAM‐C favors up to four glucose units for its catalytic activity, and this isoform is the most active of all the maltase enzymes; thus, its inhibition is a promising target for significant improvements in diabetic patients. Upon inspection of the −1‐subsite binding with angulasaponin C, His1584 and Asp1529 formed two H bonds with the OH groups of ring I. While the +2‐subsite encompassed polar interaction between Trp1369 and the OH group in ring H, the +3‐subsite showed two hydrogen bonds between Phe1560 and both the pyranosyl oxygen and OH group. Angulasaponin C superseded acarbose through Lys1164 interaction with the methyl hydroxy group of the 1,2‐linked diglucosides and Asp1532 with double hydroxy groups of the terminal ring. Clearly, these ionic interactions stabilized angulasaponin C to have a score of −13.103 Kcal/mol. Angulasaponin B encountered the same orientation in the binding subsites −1, +1, and +3 and was able to extend hydrogen bonds in the distal subsite to Lys1164 and Asp1532, yet it lacked the hydrogen bond formed with Trp1369 in the +2‐subsite, justifying its lower binding score. Angulasaponin VI and IV marginally outperformed acarbose in their binding stability. Adzukisaponin IV resulted in a lower binding score due to the single glucoside unit attachment in position 3. Interactions were summarized as follows: in the distal subsite, hydrogen bonds were formed between Lys1164 and COOH group in ring A, between Tyr1162 and the methyl hydroxy group in ring F, and between Asp1532 and the two hydroxy groups in ring F. Whereas the −1 subsite was marked by His1584 ionic interaction with OH group of ring H, the +1 subsite showed Asp1526 interacting with OH group of ring F, and the +3 subsite encompassing aromatic hydrogen bond formed between Phe1560 and the ester linking group. Adzukisaponin VI ring G methyl OH group extended hydrogen bond to Asp1526 in the −1 subsite. In the same vein, Arg1510 and Asp1157 interacted with the OH group of ring G. In the front edge of the site, Ser1452 and Arg1156 interacted with the COOH and OH groups of ring H, respectively, and Phe1247 formed ionic interaction with the methyl OH group of ring I. These interactions permitted enough fitting for adzukisaponin VI in the enzyme pocket with a score of −12.551 Kcal/mol since the formation of hydrogen bonds within the region of hydrophobic contacts is of significant impact on good binding and fitting scores. Upon conducting the molecular dynamics (MD) study of the angulasaponin C complex with maltase enzyme, the consistency of all the ionic hydrogen bond interactions seen was evident as in the docking protocol throughout the whole simulation time, namely with Arg1156, Tyr1251, Trp1355, Trp1369, Phe1427, Arg1510, and Thr1586. The stable RMSD values revolved between 2 and 2.25 A° upon binding of the ligand, indicating the conformational uniformity of molecular interactions and their contributions to the favorable binding free energy (Figures S3 and S4).

### 4.3 α‐Glucosidases

α‐glucosidases are of notable importance in obesity and type 2 diabetes mellitus where they act to release α‐glucose subunits from di‐, oligo‐, or aryl glucosides. The native ligand acarbose was used to validate the docking protocol in Maestro 11.0 where the co‐crystallized ligand was removed and redocked with an RMSD value of 1.3756 A°. Many of the screened saponins outperformed acarbose, such as adzukisaponin IV, adzukisaponin VI, angulasaponin B, and angulasaponin C, whose Glide XP scores were −11.585, −11.173, −10.285, and −11.335 Kcal/mol, respectively. The negative binding scores denoted favorable interactions with the key amino acids. GAA is a hydrolase assuring the breakage of both α‐1,4‐ and α‐1,6‐glycosidic linkages within the lysosomes (Table 4). 5nn8 is a recombinant GAA protein with particular specificity directed to the hydrolysis of α‐1,4‐glucosidic linkage 32‐fold than the α‐1,6‐link (Roig‐Zamboni et al., 2017). The structure of the enzyme was previously illustrated as a typical GH311 hydrolase protein with major similarity to the maltase‐glucoamylase (MGAM) and the sucrase‐isomaltase (SI), which are the core starch‐digesting enzymes within the intestinal border. While the maltose is formed of two glucose units joined by an α 1–4 bond, isomaltose is two glucose units linked through an α 1–6 bond. Catalytic reactions held in family GH311 enzymes retain the configuration of the anomeric proton and are of the Koshland double displacement reaction type, with Asp518and Asp616 playing the nucleophilic role and acid/base catalysis.

The binding subsites +2 and +3 were envisaged for their stabilizing role in binding of the tetrasaccharide ligands compared to the disaccharide counterparts, although their exact amino acid pattern was not clear. The reversed orientation of adzukisaponin IV, such that the single glucose unit fits into the +1 subsite, largely impacted its binding stability and interactions, which further confirmed that the α‐1,6‐attached diglucoside in the +1 and −1 subsites was essential to manifest the lowest fitting score (Figure S13). Adzukisaponins bound with a terminal nonreducing end. While acarbose showed seven hydrophilic interactions in the α‐rhGAA groove with Asp282 (1.93, 2.33, 2.11), Asp616 (2.23,1.71), and Asp404 (1.95), it was close enough to Phe525 to extend a hydrophobic contact at 2.11A°. The hydrophilic nature of the ligands appears to contribute to a better binding score, which is manifested form inspecting angulasaponin A (−7 Kcal/mol) and adzukisaponin I (−7.05 Kcal/mol) whose skeletons encompass only one disaccharide unit, instead of two, in the better binding compounds. Moreover, the larger size of adzukisaponins, due to the triterpenoidal link, helped to provide a better orientation and interaction distances with amino acids than acarbose. Adzukisaponin IV and VI interacted with the same polar residues as acarbose, namely, Asp404, asp616, and Asp282; additionally, their better binding scores could be attributed to their larger number of ionic interactions with Arg600 and the salt bridge formation with Arg411 and Lys470, respectively. The hydrophobic contacts with Trp481 and Val480 might have contributed to a stable binding mode than acarbose (Figure S13). On the other hand, angulasaponins A and D recorded lower number of hydrogen bond interactions and lacked binding to Asp404 and Asp616, respectively, which largely compromised their Glide XP scores to be less than acarbose. Angulasaponins B and C were superior to acarbose, despite lacking polar interactions with key amino acids as Asp404 and Asp282, but this was compensated by their hydrogen bonds to Asp518 and Arg600 as well as the salt bridge formed between Lys479 and angulasaponin B or between Pro482 and angulasaponin C.

Adzukisaponin IV complex with α‐glucosidase revealed constant interactions with Trp376, Arg411, and Trp481 during the simulation study. The trajectory analysis of both the apo‐α‐glucosidase enzyme and its bounded form manifested homogeneity in their behavior through the 100 ns simulation time, as seen in the plateau formed upon investigating the RMSD within a range between 1.5 and 2A. Similarly, the RMSF values of the sampled amino acids showed little fluctuations with or without a bounded ligand, which further confirmed the stability of the α ‐glucosidase–adzukisaponin IV complex and its promising potential as anti‐obesity molecule (Figures S5 and S6).

### 4.4 α‐Amylases

In order to assess the binding mode, energy, and interaction of the selected ligands inside the pancreatic‐α‐amylase enzyme (4 W93), molecular docking was performed and validated with MD simulations and free energy calculations. Three core amino acids featured the conserved catalytic residues in α‐amylase, Glu 233, Asp 197, and Asp 300 (Williams et al., 2015). Upon inspecting the 2D and 3D interactions of adzukisaponins inside the binding groove, these residues were detected forming clear ionic hydrogen bond interactions both directly and mediated by water molecules intrinsic to the protein pocket (Guan et al., 2022).

The most stable compound with the best fitting energy score −15.599 Kcal/mol was adzukisaponin VI, assuming five hydrogen bond interactions with Glu240 (2.03, 1.92), Asp353 (2.69), Asp356 (1.70), and Asp300 (1.70). The highest Glide XP scores were achieved by adzukisaponin VI, angulasaponin B, and angulasaponin C whose structures possessed four sugar units attached as two maltose units in positions 3 and 29 of the tritepenoidal skeleton (Table 3). While the hydrophobic bed was effectively filled with the triterpenoidal skeleton, which revealed close contact to Trp357, Val354, Tyr151, Leu162, Ile235, Gly306, Thr163, Leu237, Trp58, and Trp59, the polar side pockets were clearly interacting with the disaccharide units of adzukisaponin VI, with an emphasis on the importance of binding distances, and the number of hydrogen bonds, polar and nonpolar contacts (Figure S14).

Angulasaponin B displayed obvious hydrogen bonds with the conserved pair of amino acids, Asp197 (1.64) and Glu233 (2.62), impeded within hydrophobic patches formed of Trp269 and Ala307, Glu272, which strengthen their rewarding energy; moreover, a salt bridge was formed between the carboxylic group in ring G and Lys261 (3.02) Table 2. The native ligand montbretin conferred a better fitting to the enzyme core through its π‐π‐stacking interactions with Tyr62 and His290. A salt bridge was noted between Lys200 and the carboxylate group of the galacturonic acid unit, which contributed to the better binding conferred by adzukisaponin VI, even better than the standard drug acarbose (−13.082 Kcal/mol) (Figures S15 and S16). The pancreatic α‐amylase‐binding site is characterized by key aspartate amino acids Asp300, Asp353, and Asp356 and the π‐π‐stacking interaction with residues Tyr62 and His290. The docking procedures were validated by redocking the montbretin A into the active site with an RMSD value of 0.0221 A° (Figure 2). The selected saponin compounds were bound in the active site of α‐amylase assuming the same direction and orientation of the co‐crystallized ligand montbretin A.

Upon conducting extensive analysis using molecular dynamics (MD) simulation for 100 ns, the adzukisaponin VI–amylase complex manifested stability of its binding interactions throughout most of the simulation time (Figure S7). This was evident by investigating the Rg analysis for both the free α‐amylase and its bounded state trajectories. The RMSF analysis, which measured the displacement degree of atoms compared to reference structures, again confirmed the stability of adzukisaponin VI complex in the range 23–23.2 Å than the angulasaponin B complex whose loop region fluctuated till 23.7 A at 60 ns. The RMSD measured the conformational fluctuation of a protein during the simulation from its initial position to its final conformation. The trajectory of the dynamics analysis revealed the continuous interaction of adzukisaponin VI with Asp353, Glu240, and Lys200, as shown in the amino acid contacts in (Figure S8). The angulasaponin B–α‐amylase complex manifested stable and consistent interactions with Asp197, Glu233, and Thr264 with other residues as Trp59 and Leu165 (Figure S13).

The free binding energy calculations under physiological conditions denoted as the molecular mechanics Generalized Born Surface Area (MMGBSA) where water was selected as a solvent to compute the energy terms GBSA or generalized born surface area was another method to assess the stability of ligand interactions with the binding pocket. Upon visualizing the GBSA energy components at the beginning and the end of simulation, it was evident that the energy ranges were very close to each other, suggesting the binding stability and consistency during the simulation run and thus the potency and efficacy of these ligands (Figure S9; Table 2).

## 5 CONCLUSIONS

Herein, we computationally studied the antidiabetic and anti‐obesity effects of the popular food component, *V. angularis*. A selected compounds library was utilized encompassing 10 saponin triterpenoids to assess the validity of the hypothesis that saponins of *V. angularis* rather than polyphenolics contribute to the weight‐reducing effect of adzuki beans. Molecular docking results showed the superior fundamental interactions presented by adzukisaponin VI, adzukisaponin IV, and angulasaponins B and C, even surpassing the control drug acarbose and native ligands of the carbohydrate‐digesting enzymes, pancreatic α‐amylase, α‐glucosidase, intestinal sucrase, and intestinal maltase‐glucoamylase. The stability of the protein–ligand complexes was assessed through MD trajectories during 100 ns by calculating the RMSD, RMSF, and the Rg values to indicate the consistency of binding to important amino acid residues and the absence of unfolding. The two complexes, maltase–aduzkisaponin VI complex and α‐amylase with angulasaponin B, revealed MMGBSA free energy calculations of −67.77 and −74.18 Kcal/mol, respectively, with the least change from the start to the end of the simulation time. The use of adzuki beans saponins warrants more experimental in vitro and in vivo studies to clarify the mechanism of its natural anti‐obesity effect.

## AUTHOR CONTRIBUTIONS

**Ashaimaa Y. Moussa:** Conceptualization (equal); data curation (equal); formal analysis (equal); investigation (equal); methodology (equal); software (equal); validation (equal); visualization (equal); writing – original draft (equal). **Abdullah Alanzi:** Data curation (equal); formal analysis (equal); methodology (equal); software (equal); validation (equal); visualization (equal); writing – original draft (equal). **Jinhai Luo:** Data curation (equal); investigation (equal); methodology (equal); resources (equal); validation (equal); writing – original draft (equal). **Sookja Kim Chung:** Funding acquisition (equal); project administration (equal); resources (equal); validation (equal); writing – review and editing (equal). **Baojun Xu:** Conceptualization (equal); funding acquisition (equal); project administration (equal); resources (equal); supervision (equal); validation (equal); visualization (equal); writing – review and editing (equal).

## FUNDING INFORMATION

This research was jointly funded by two funds from the National Natural Science Foundation of China (Project code: 32172198 and 81771353) and a Researcher's Supporting Project (Grant No. RSPD2024R885) from King Saud University (Riyadh, Saudi Arabia).

## CONFLICT OF INTEREST STATEMENT

The authors declare no conflict of interest.

## Supporting information


File S1.

## Data Availability

The data that support the findings of this study are available on request from the corresponding author.

## References

[fsn34032-bib-0001] AbdelRazek, M. M. , Elissawy, A. M. , Mostafa, N. M. , Moussa, A. Y. , Elanany, M. A. , Elshanawany, M. A. , & Singab, A. N. B. (2023). Chemical and biological review of endophytic fungi associated with Morus sp.(Moraceae) and in silico study of their antidiabetic potential. Molecules, 28(4), 1718. 10.3390/molecules28041718 36838706 PMC9968060

[fsn34032-bib-0002] Al‐Goblan, A. S. , Al‐Alfi, M. A. , & Khan, M. Z. (2014 Dec). Mechanism linking diabetes mellitus and obesity. Diabetes Metab Syndr Obes., 4(7), 587–591. 10.2147/DMSO.S67400 PMC4259868 25506234

[fsn34032-bib-0003] Ashraf, H. , Moussa, A. , Seleem, A. , Eldahshan, O. A. , & Singab, A.‐N. (2020). UPLC‐ESI/MS/MS profiling and anti‐inflammatory activity of Gleditsia caspica. Archives of Pharmaceutical Sciences Ain Shams University, 4(1), 124–134. 10.21608/aps.2020.2004.1042

[fsn34032-bib-0004] Ashraf, H. , Moussa, A. Y. , Eldahshan, O. A. , & Singab, A. N. B. (2022). Genus Gleditsia: A phytochemical and biological review (2015‐2020). Journal of Biologically Active Products from Nature, 12(1), 1–23. 10.1080/22311866.2021.2013943

[fsn34032-bib-0005] Case, D. A. , Aktulga, H. M. , Belfon, K. , Ben‐Shalom, I. , Brozell, S. R. , Cerutti, D. S. , Cheatham, T. E., III , Cruzeiro, V. W. D. , Darden, T. A. , & Duke, R. E. (2021). Amber 2021. University of California.

[fsn34032-bib-0006] Case, D. A. , Cheatham, T. E., III , Darden, T. , Gohlke, H. , Luo, R. , Merz, K. M., Jr. , Onufriev, A. , Simmerling, C. , Wang, B. , & Woods, R. J. (2005). The Amber biomolecular simulation programs. Journal of Computational Chemistry, 26(16), 1668–1688. 16200636 10.1002/jcc.20290 PMC1989667

[fsn34032-bib-0007] Duan, Y. , Wu, C. , Chowdhury, S. , Lee, M. C. , Xiong, G. , Zhang, W. , Yang, R. , Cieplak, P. , Luo, R. , & Lee, T. (2003). A point‐charge force field for molecular mechanics simulations of proteins based on condensed‐phase quantum mechanical calculations. Journal of Computational Chemistry, 24(16), 1999–2012. 10.1002/jcc.20290 14531054

[fsn34032-bib-0008] Fayez, S. , Ayoub, I. M. , Mostafa, N. M. , Moussa, A. Y. , Gamal ElDin, M. I. , & El‐Shazly, M. (2022). Nutraceuticals in cancer therapy. In S. Chakraborti (Ed.), Handbook of oxidative stress in cancer: Therapeutic aspects. Springer. 10.1007/978-981-16-5422-0_15

[fsn34032-bib-0101] Gan, R. , Wang, M. , Lui, W. , Wu, K. , & Corke, H. (2016). Dynamic changes in phytochemical composition and antioxidant capacity in green and black Mung Bean (*Vigna radiata*) sprouts. International Journal of Food Science & Technology, 51(9), 2090–2098. 10.1111/ijfs.13185

[fsn34032-bib-0009] Grant, B. J. , Skjærven, L. , & Yao, X.‐Q. (2021). The Bio3D packages for structural bioinformatics. Protein Science, 30(1), 20–30. 10.1002/pro.3923 32734663 PMC7737766

[fsn34032-bib-0010] Guan, L. , Long, H. , Ren, F. , Li, Y. , & Zhang, H. (2022). A structure—Activity relationship study of the inhibition of α‐amylase by benzoic acid and its derivatives. Nutrients, 14(9), 1931. 10.3390/nu14091931 35565898 PMC9102017

[fsn34032-bib-0011] Guo, L. , Gao, Z. , Zhang, L. , Guo, F. , Chen, Y. , Li, Y. , & Huang, C. (2016). Saponin‐enriched sea cucumber extracts exhibit an antiobesity effect through inhibition of pancreatic lipase activity and upregulation of LXR‐β signaling. Pharmaceutical Biology, 54(8), 1312–1325. 10.3109/13880209.2015.1075047 26440226

[fsn34032-bib-0013] Hyde, P. N. , Sapper, T. N. , Crabtree, C. D. , Lafountain, R. A. , Bowling, M. L. , Buga, A. , Fell, B. , McSwiney, F. T. , Dickerson, R. M. , Miller, V. J. , Scandling, D. , Simonetti, O. P. , Phinney, S. D. , Kraemer, W. J. , King, S. A. , Krauss, R. M. , & Volek, J. S. (2019). Dietary carbohydrate restriction improves metabolic syndrome independent of weight loss. JCI Insight, 4(12), e128308. 31217353 10.1172/jci.insight.128308 PMC6629108

[fsn34032-bib-0014] Iida, T. , Yoshiki, Y. , Kahara, T. , Okubo, K. , & Ohrui, H. (1997). A saponin conjugated with 2, 3‐dihydro‐2, 5‐dihydroxy‐6‐methyl‐4H‐pyran‐4‐one from *Vigna angularis* . Phytochemistry, 45(7), 1507–1509. 10.1016/s0031-9422(97)00169-6

[fsn34032-bib-0015] Iida, T. , Yoshiki, Y. , Okubo, K. , Ohrui, H. , Kinjo, J. , & Nohara, T. (1999). Triterpenoid saponins from *Vigna angularis* . Phytochemistry, 51(8), 1055–1058. 10.1016/s0031-9422(99)00148-x

[fsn34032-bib-0016] Itoh, T. , Kita, N. , Kurokawa, Y. , Kobayashi, M. , Horio, F. , & Furuichi, Y. (2004). Suppressive effect of a hot water extract of adzuki beans (*Vigna angularis*) on hyperglycemia after sucrose loading in mice and diabetic rats. Bioscience, Biotechnology, and Biochemistry, 68(12), 2421–2426. 10.1271/bbb.68.2421 15618610

[fsn34032-bib-0102] Itoh, T. , Kobayashi, M. , Horio, F. , & Furuichi, Y. (2009). Hypoglycemic effect of hot‐water extract of adzuki (*Vigna angularis*) in spontaneously diabetic KK‐Ay mice. Nutrition, 25(2), 134–141. 10.1016/j.nut.2008.08.001 18929464

[fsn34032-bib-0017] Jiang, Y. , Zeng, K.‐W. , David, B. , & Massiot, G. (2014). Constituents of *Vigna angularis* and their in vitro anti‐inflammatory activity. Phytochemistry, 107, 111–118. 10.1016/j.phytochem.2014.08.011 25189119

[fsn34032-bib-0018] Kawahara, S.‐I. , Ishihara, C. , Matsumoto, K. , Senga, S. , Kawaguchi, K. , Yamamoto, A. , Suwannachot, J. , Hamauzu, Y. , Makabe, H. , & Fujii, H. (2019). Identification and characterization of oligomeric proanthocyanidins with significant anti‐cancer activity in adzuki beans (*Vigna angularis*). Heliyon, 5(10), e02610. 10.1016/j.heliyon.2019.e02610 31687492 PMC6820087

[fsn34032-bib-0019] Kawakami, W. , Oshima, A. , & Yanase, E. (2018). Structural characterization of proanthocyanidins from adzuki seed coat. Food Chemistry, 239, 1110–1116. 10.1016/j.foodchem.2017.07.001 28873529

[fsn34032-bib-0020] Kim, M. , Kim, D. K. , & Cha, Y.‐S. (2016). Black adzuki bean (*Vigna angularis*) extract protects pancreatic β cells and improves glucose tolerance in C57BL/6J mice fed a high‐fat diet. Journal of Medicinal Food, 19(5), 442–449. 10.1089/jmf.2015.3598 27070495

[fsn34032-bib-0021] Kim, M. , Park, J.‐E. , Song, S.‐B. , & Cha, Y.‐S. (2015). Effects of black adzuki bean (*Vigna angularis*) extract on proliferation and differentiation of 3T3‐L1 preadipocytesinto mature adipocytes. Nutrients, 7(1), 277–292. 10.3390/nu7010277 25569623 PMC4303839

[fsn34032-bib-0022] Kitano‐Okada, T. , Ito, A. , Koide, A. , Nakamura, Y. , Han, K. H. , Shimada, K. , Sasaki, K. , Ohba, K. , Sibayama, S. , & Fukushima, M. (2012). Anti‐obesity role of adzuki bean extract containing polyphenols: In vivo and in vitro effects. Journal of the Science of Food and Agriculture, 92(13), 2644–2651. 10.1002/jsfa.5680 22495778

[fsn34032-bib-0023] Kojima, M. , Ohnishi, M. , Ito, S. , & Fujino, Y. (1989). Characterization of acylmono‐, mono‐, di‐, tri‐and tetraglycosylsterol and saponin in adzuki bean (*Vigna angularis*) seeds. Lipids, 24, 849–853. 10.1007/bf02535758 27520439

[fsn34032-bib-0024] Kris‐Etherton, P. M. , Hecker, K. D. , Bonanome, A. , Coval, S. M. , Binkoski, A. E. , Hilpert, K. F. , Griel, A. E. , & Etherton, T. D. (2002). Bioactive compounds in foods: Their role in the prevention of cardiovascular disease and cancer. The American Journal of Medicine, 113(9), 71–88. 10.1016/S0002-9343(01)00995-0 12566142

[fsn34032-bib-0103] Kuriya, B. , Schieir, O. , Valois, M. F. , Pope, J. E. , Boire, G. , Bessette, L. , Hazlewood, G. , Thorne, J. C. , Tin, D. , Hitchon, C. , Bartlett, S. J. , Keystone, E. C. , Bykerk, V. P. , & Barra, L. (2019). Prevalence and characteristics of metabolic syndrome differ in men and women with early rheumatoid arthritis. ACR Open Rheumatology, 1(9), 535–541. 10.1002/acr2.11075 31777836 PMC6858015

[fsn34032-bib-0025] Lee, P. S. , Teng, C. Y. , Hsieh, K. F. , Chiou, Y. S. , Wu, J. C. , Lu, T. J. , & Pan, M. H. (2019). Adzuki bean water extract attenuates obesity by modulating M2/M1 macrophage polarization and gut microbiota composition. Molecular Nutrition & Food Research, 63(23), 1900626. 10.1002/mnfr.201900626 31574574

[fsn34032-bib-0026] Li, W. , Song, Y. , Cao, Y.‐N. , Zhang, L.‐L. , Zhao, G. , Wu, D.‐T. , & Zou, L. (2022). Total saponins from quinoa bran alleviate high‐fat diet‐induced obesity and systemic inflammation via regulation of gut microbiota in rats. Food Science & Nutrition, 10, 3876–3889. 10.1002/fsn3.2984 36348812 PMC9632199

[fsn34032-bib-0027] Liu, R. , & Xu, B. (2016). Bioactive compositions and health promoting effects of adzuki bean. Phytotherapeutics‐III, 44, 23–43.

[fsn34032-bib-0028] Liu, R. , Zheng, Y. , Cai, Z. , & Xu, B. (2017). Saponins and flavonoids from adzuki bean (*Vigna angularis* L.) ameliorate high‐fat diet‐induced obesity in ICR mice. Frontiers in Pharmacology, 8, 687. 10.3389/fphar.2017.00687 29021760 PMC5623717

[fsn34032-bib-0029] Luo, J. , Cai, W. , Wu, T. , & Xu, B. (2016). Phytochemical distribution in hull and cotyledon of adzuki bean (*Vigna angularis* L.) and mung bean (*Vigna radiate* L.), and their contribution to antioxidant, anti‐inflammatory and anti‐diabetic activities. Food Chemistry, 201, 350–360. 10.1016/j.foodchem.2016.01.101 26868587

[fsn34032-bib-0030] Maruyama, C. , Araki, R. , Kawamura, M. , Kondo, N. , Kigawa, M. , Kawai, Y. , Takanami, Y. , Miyashita, K. , & Shimomitsu, T. (2008). Azuki bean juice lowers serum triglyceride concentrations in healthy young women. Journal of Clinical Biochemistry and Nutrition, 43(1), 19–25. 10.3164/jcbn.2008039 18648655 PMC2459248

[fsn34032-bib-0031] Moussa, A. Y. (2013). Isolation of chemical constituents and protective effect of Pistacia khinjuk against CCl4‐induced damage on HepG2 cells. Phytopharmacology, 4, 1–9.

[fsn34032-bib-0032] Moussa, A. Y. , Mostafa, N. M. , & Singab, A. N. B. (2020). Pulchranin a: First report of isolation from an endophytic fungus and its inhibitory activity on cyclin dependent kinases. Natural Product Research, 34(19), 2715–2722. 10.1080/14786419.2019.1585846 30887847

[fsn34032-bib-0033] Newman, D. J. (2021). Problems that can occur when assaying extracts to pure compounds in biological systems. Current Therapeutic Research, 95, 100645. 10.1016/j.curtheres.2021.100645 34691294 PMC8515388

[fsn34032-bib-0104] Nishi, S. , Saito, Y. , Souma, C. , Kato, J. , Koaze, H. , Hironaka, K. , & Kojima, M. (2008). Suppression of serum cholesterol levels in mice by Adzuki Bean Polyphenols. Food Science and Technology Research, 14(2), 217–220. 10.3136/fstr.14.217

[fsn34032-bib-0034] Phillips, J. C. , Hardy, D. J. , Maia, J. D. , Stone, J. E. , Ribeiro, J. V. , Bernardi, R. C. , Buch, R. , Fiorin, G. , Hénin, J. , & Jiang, W. (2020). Scalable molecular dynamics on CPU and GPU architectures with NAMD. The Journal of Chemical Physics, 153(4), 44130. 10.1063/5.0014475 PMC7395834 32752662

[fsn34032-bib-0035] Qian, Q. , Zhang, Z. , Li, M. , Savage, K. , Cheng, D. , Rauckhorst, A. J. , Ankrum, J. A. , Taylor, E. B. , Ding, W. X. , Xiao, Y. , Cao, H. J. , & Yang, L. (2019). Hepatic lysosomal iNOS activity impairs autophagy in obesity. Cell Mol Gastroenterología y Hepatología, 8(1), 95–110. 10.1016/j.jcmgh.2019.03.005 PMC6522853 30926581

[fsn34032-bib-0036] Ren, L. , Qin, X. , Cao, X. , Wang, L. , Bai, F. , Bai, G. , & Shen, Y. (2011). Structural insight into substrate specificity of human intestinal maltase‐glucoamylase. Protein & Cell, 2, 827–836. 10.1007/s13238-011-1105-3 22058037 PMC4875297

[fsn34032-bib-0037] Rodrigues, T. (2019). The good, the bad, and the ugly in chemical and biological data for machine learning. Drug Discovery Today: Technologies, 32, 3–8. 10.1016/j.ddtec.2020.07.001 33386092 PMC7382642

[fsn34032-bib-0038] Roig‐Zamboni, V. , Cobucci‐Ponzano, B. , Iacono, R. , Ferrara, M. C. , Germany, S. , Bourne, Y. , Parenti, G. , Moracci, M. , & Sulzenbacher, G. (2017). Structure of human lysosomal acid α‐glucosidase–a guide for the treatment of Pompe disease. Nature Communications, 8(1), 1111. 10.1038/s41467-017-01263-3 PMC5653652 29061980

[fsn34032-bib-0039] Sansbury, B. E. , & Hill, B. G. (2014). Regulation of obesity and insulin resistance by nitric oxide. Free Radical Biology and Medicine, 73, 383–399. 10.1016/j.freeradbiomed.2014.05.016 24878261 PMC4112002

[fsn34032-bib-0040] Shi, Z. , Zhu, Y. , Teng, C. , Yao, Y. , Ren, G. , & Richel, A. (2020). Anti‐obesity effects of α‐amylase inhibitor enriched‐extract from white common beans (Phaseolus vulgaris L.) associated with the modulation of gut microbiota composition in high‐fat diet‐induced obese rats. Food & Function, 11(2), 1624–1634. 10.1039/c9fo01813a 32022058

[fsn34032-bib-0041] Sim, L. , Willemsma, C. , Mohan, S. , Naim, H. Y. , Pinto, B. M. , & Rose, D. R. (2010). Structural basis for substrate selectivity in human maltase‐glucoamylase and sucrase‐isomaltase N‐terminal domains. Journal of Biological Chemistry, 285(23), 17763–17770. 10.1074/jbc.m109.078980 20356844 PMC2878540

[fsn34032-bib-0042] Sreerama, Y. N. , Takahashi, Y. , & Yamaki, K. (2012). Phenolic antioxidants in some Vigna species of legumes and their distinct inhibitory effects on α‐glucosidase and pancreatic lipase activities. Journal of Food Science, 77(9), C927–C933. 10.1111/j.1750-3841.2012.02848.x 22889371

[fsn34032-bib-0043] Torky, Z. A. , Moussa, A. Y. , Abdelghffar, E. A. , Abdel‐Hameed, U. K. , & Eldahshan, O. A. (2021). Chemical profiling, antiviral and antiproliferative activities of the essential oil of Phlomis aurea Decne grown in Egypt. Food & Function, 12(10), 4630–4643. 10.1039/d0fo03417g 33912870

[fsn34032-bib-0044] Wang, Y. , Yao, X. , Shen, H. , Zhao, R. , Li, Z. , Shen, X. , Wang, F. , Chen, K. , Zhou, Y. , & Li, B. (2022). Nutritional composition, efficacy, and processing of *Vigna angularis* (adzuki bean) for the human diet: An overview. Molecules, 27(18), 6079. 10.3390/molecules27186079 36144812 PMC9506481

[fsn34032-bib-0045] Weinzierl, R. O. (2021). Molecular dynamics simulations of human FOXO3 reveal intrinsically disordered regions spread spatially by intramolecular electrostatic repulsion. Biomolecules, 11(6), 856. 10.3390/biom11060856 34201262 PMC8228108

[fsn34032-bib-0046] William, H. (1996). VMD‐visual molecular dynamics. Journal of Molecular Graphics, 14, 33–38. 10.1016/0263-7855(96)00018-5 8744570

[fsn34032-bib-0047] Williams, L. K. , Zhang, X. , Caner, S. , Tysoe, C. , Nguyen, N. T. , Wicki, J. , Williams, D. E. , Coleman, J. , McNeill, J. H. , & Yuen, V. (2015). The amylase inhibitor montbretin a reveals a new glycosidase inhibition motif. Nature Chemical Biology, 11(9), 691–696. 10.1038/nchembio.1865 26214255

[fsn34032-bib-0105] Wu, G. , Bai, Z. , Wan, Y. , Shi, H. , Huang, X. , & Nie, S. (2020). Antidiabetic effects of polysaccharide from Azuki Bean (*Vigna angularis*) in type 2 diabetic rats via insulin/PI3K/AKT signaling pathway. Food Hydrocolloids, 101, 105456. 10.1016/j.foodhyd.2019.105456

[fsn34032-bib-0106] Yao, Y. , Cheng, X. , Wang, L. , Wang, S. , & Ren, G. (2011). A determination of potential α‐glucosidase inhibitors from Azuki Beans (*Vigna angularis*). International Journal of Molecular Sciences, 12(10), 6445–6451. 10.3390/ijms12106445 22072898 PMC3210989

[fsn34032-bib-0107] Yao, Y. , Cheng, X.‐Z. , Wang, L.‐X. , Wang, S.‐H. , & Ren, G. (2012). Major phenolic compounds, antioxidant capacity and antidiabetic potential of Rice Bean (*Vigna umbellata* L.) in China. International Journal of Molecular Sciences, 13(3), 2707–2716. 10.3390/ijms13032707 22489119 PMC3317682

