# Supplementary material for: Potential anti‐obesity effect of saponin metabolites from adzuki beans: A computational approach
Source: Food Sci Nutr. 2024 Feb 22;12(5):3612–27. doi: 10.1002/fsn3.4032 (PMC11077217; doi:10.1002/fsn3.4032)
Supplement: Supplementary file 1 — File S1. [file FSN3-12-3612-s001.docx]

**Supplementary file**

**Potential Anti-obesity Effect of Saponin Metabolites from Adzuki Beans: A Computational Approach**

**Ashaimaa Y. Moussa ^a^, Abdullah Alanzi ^b^, Jinhai Luo ^c^, Sookja Kim Chung ^d, *^, Baojun Xu ^c, *^**

*^a^* Department of Pharmacognosy, Faculty of Pharmacy, Ain Shams University, 11566, Abbasia, Cairo, Egypt; ashaimaa_yehia@pharma.asu.edu.eg.

*^b^* Department of Pharmacognosy, College of Pharmacy, King Saud University, Riyadh 11451, Saudi Arabia; [aralonazi@ksu.edu.sa](mailto:aralonazi@ksu.edu.sa).

*^c^* Food Science and Technology Program, Department of Life Sciences, BNU-HKBU United International College, Zhuhai, Guangdong 519087, China; luojinhai@uic.edu.cn; baojunxu@uic.edu.hk.

*^d^* Medical Faculty, Macau University of Science and Technology, Macau, China; skchung@must.edu.mo.

**^*^Corresponding Author at**

Prof. Baojun Xu, BNU-HKBU United International College. baojunxu@uic.edu.hk (B.X.)

**^*^Co-corresponding Author at**

Prof. Sookja Kim Chung, Macau University of Science and Technology, [skchung@must.edu.mo](mailto:skchung@must.edu.mo) (S.K.C.)


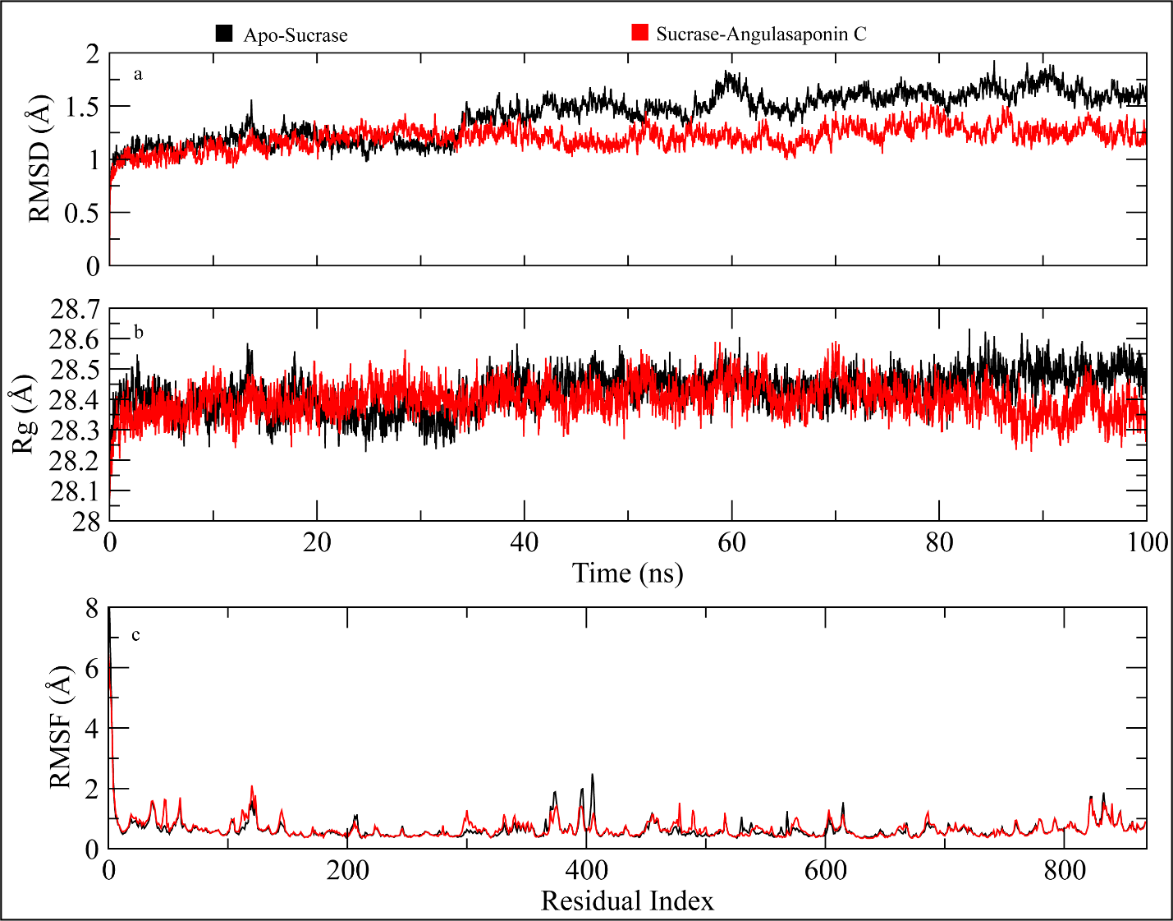
**Figure 1S.** The protein-ligand stability analysis of sucrase-angulasaponin C complex. (a) The RMSD of protein backbone atoms. (b) The Rg plot of the protein to analyze the compactness. (c) The RMSF of the protein residues.


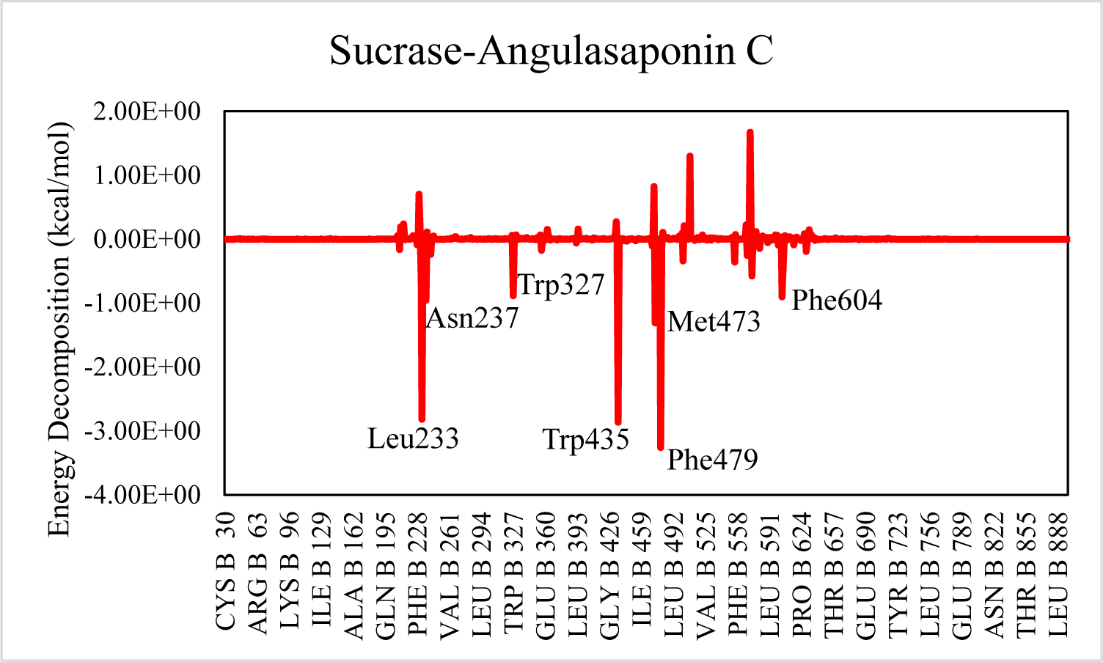


**Figure 2S.** The protein-ligand contacts of sucrase-angulasaponin C complex.


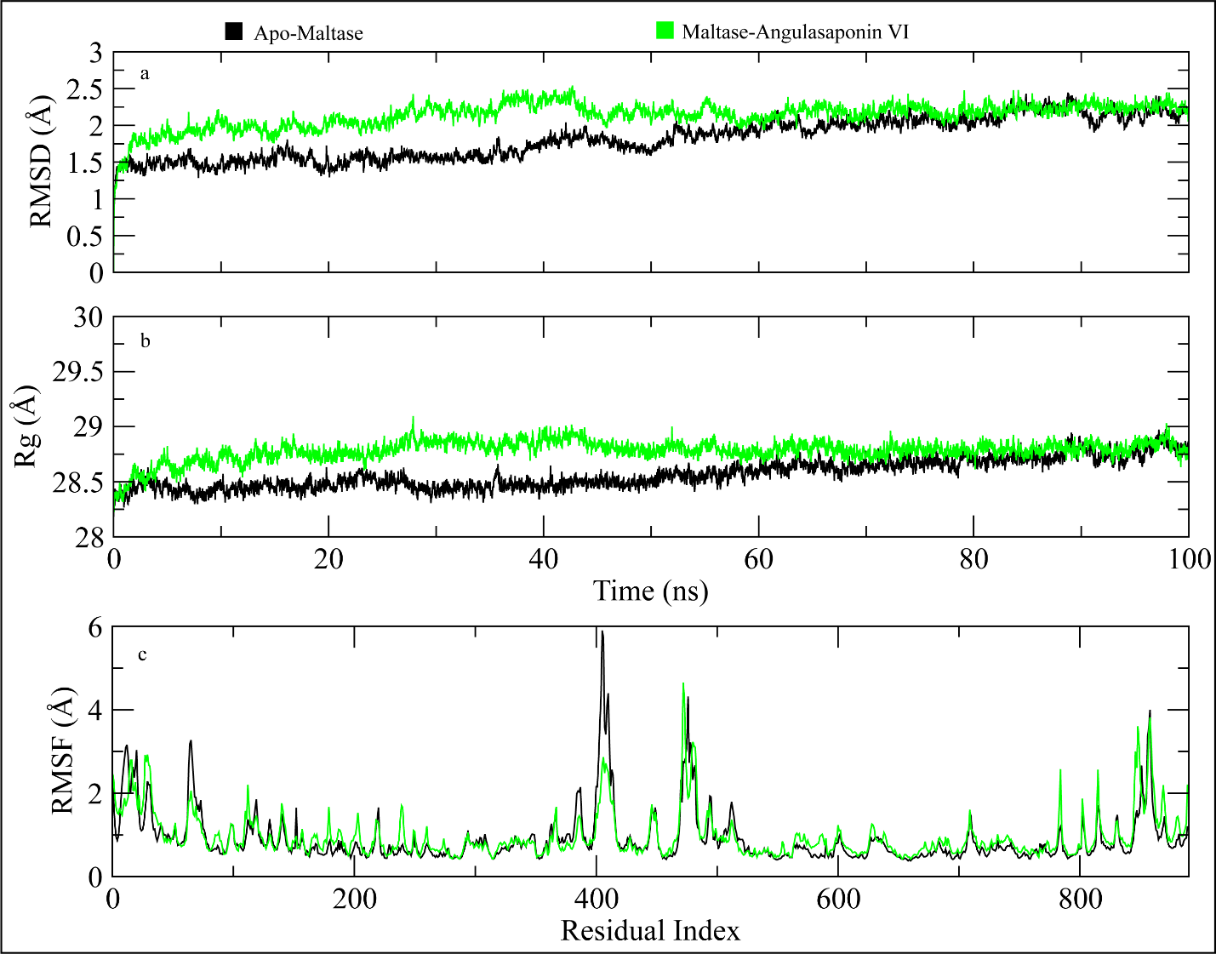
**Figure 3S.** The protein-ligand stability analysis of maltase-angulasaponin VI complex. (a) The RMSD of protein backbone atoms. (b) The Rg plot of the protein to analyze the compactness. (c) The RMSF of the protein residues.


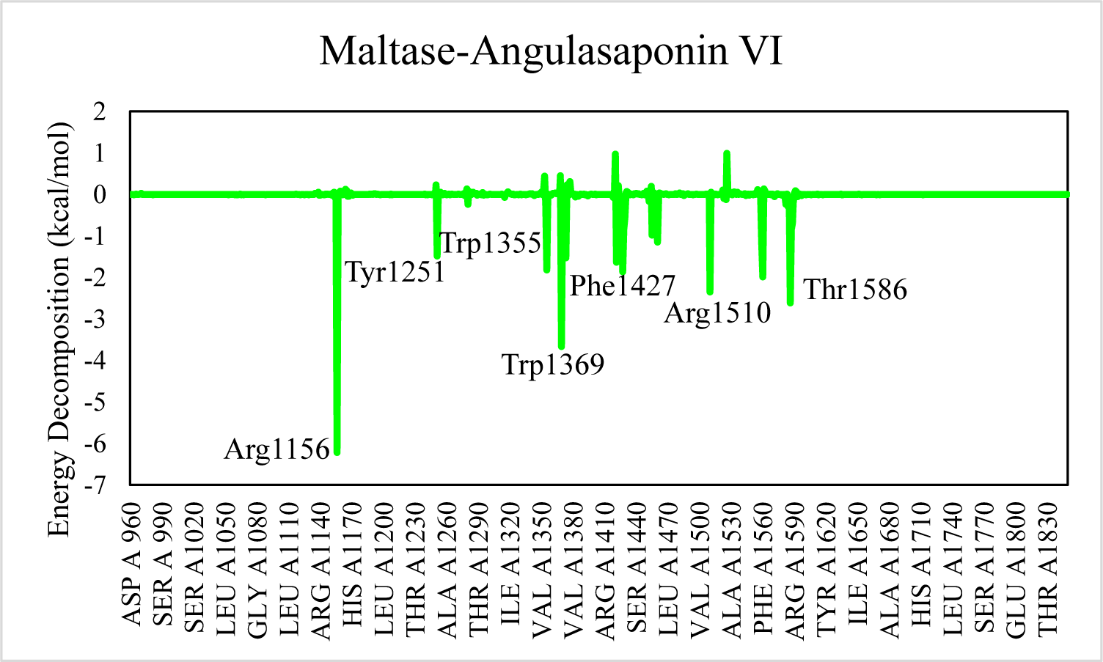


**Figure 4S.** The protein-ligand amino acid contacts of maltase-angulasaponin VI complex.


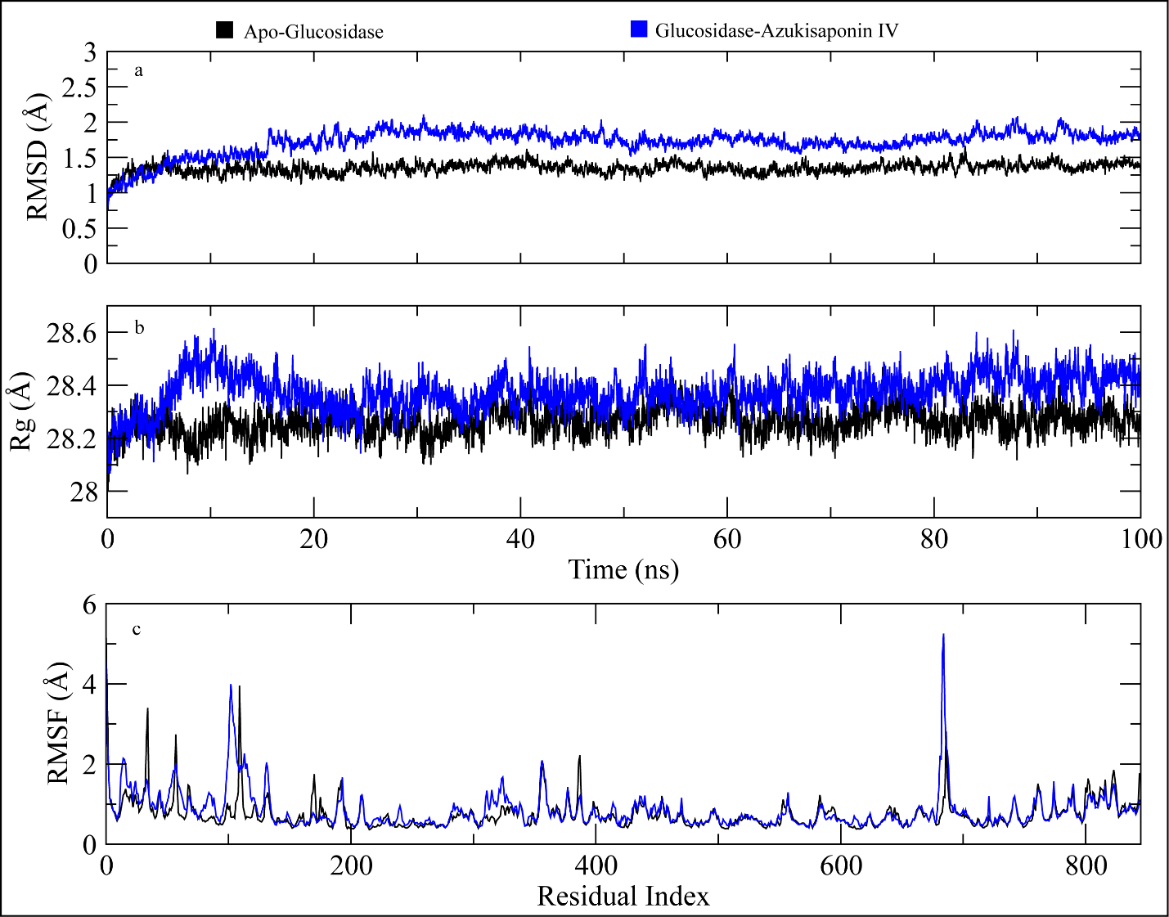
**Figure 5S**. The protein-ligand stability analysis of α-glucosidase- azukisaponin IV complex. (a) The RMSD of protein backbone atoms. (b) The Rg plot of the protein to analyze the compactness. (c) The RMSF of the protein residues.


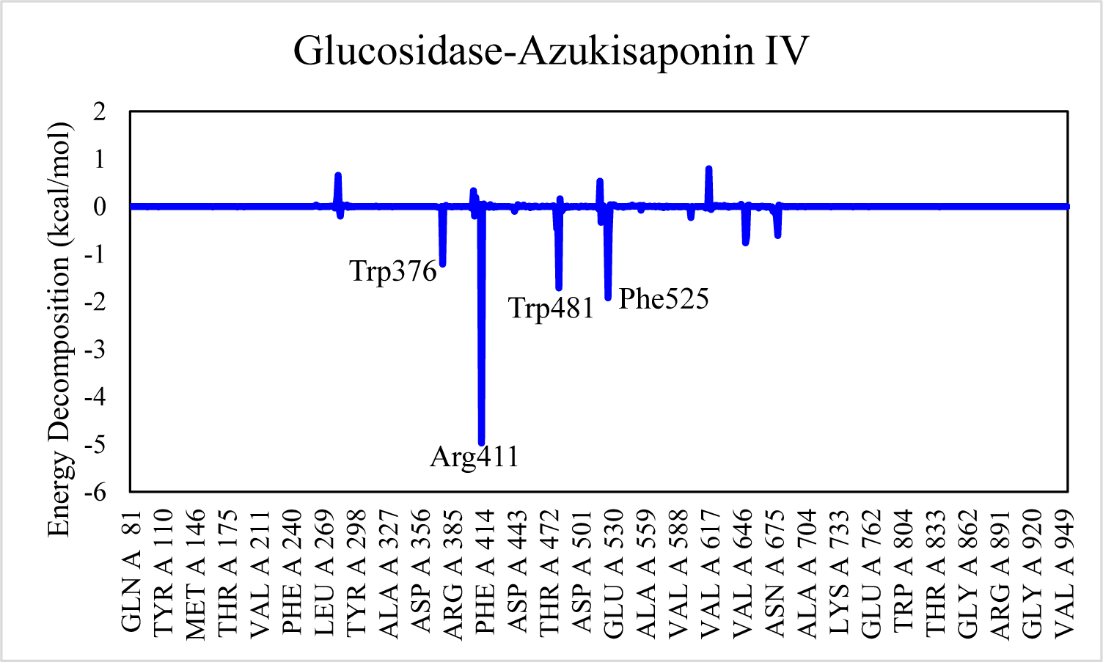
**Figure 6S**. The protein-ligand amino acid contacts of α-glucosidase- adzukisaponin IV com- plex.


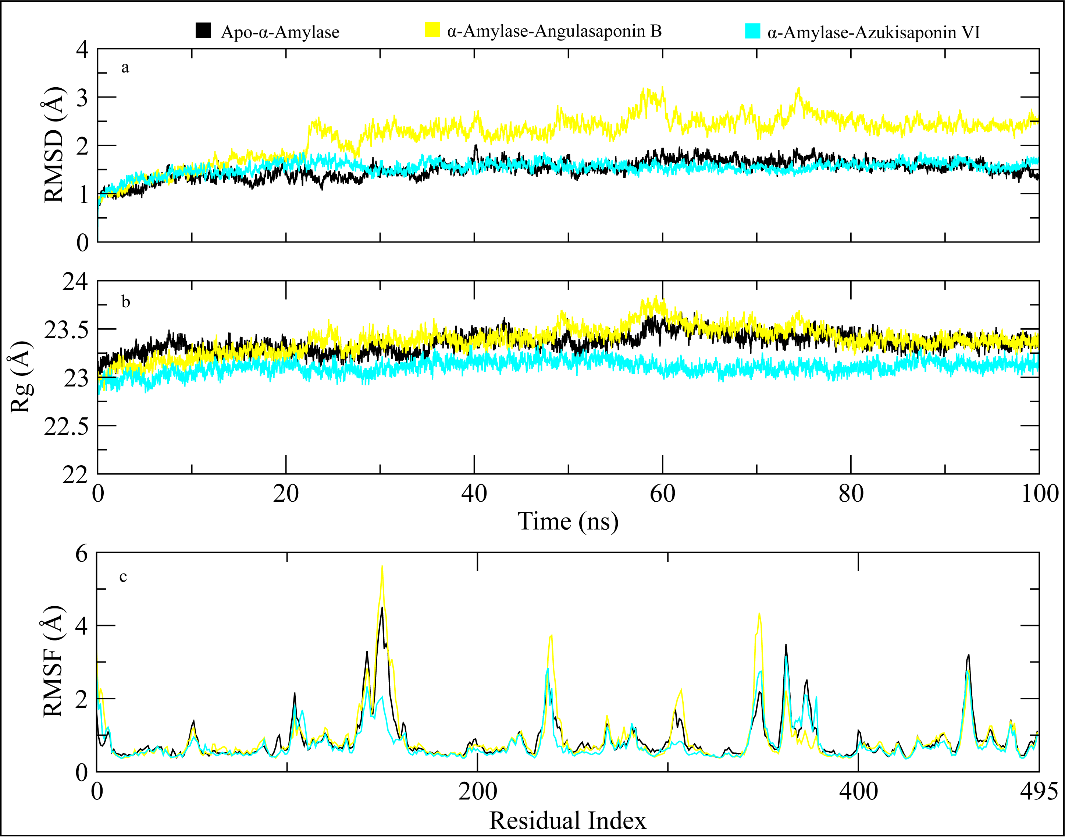
**Figure 7S.** The protein-ligand stability analysis of α-amylase complexes. (a) The RMSD of protein backbone atoms. (b) The Rg plot of the protein to analyze the compactness. (c) The RMSF of the protein residues.


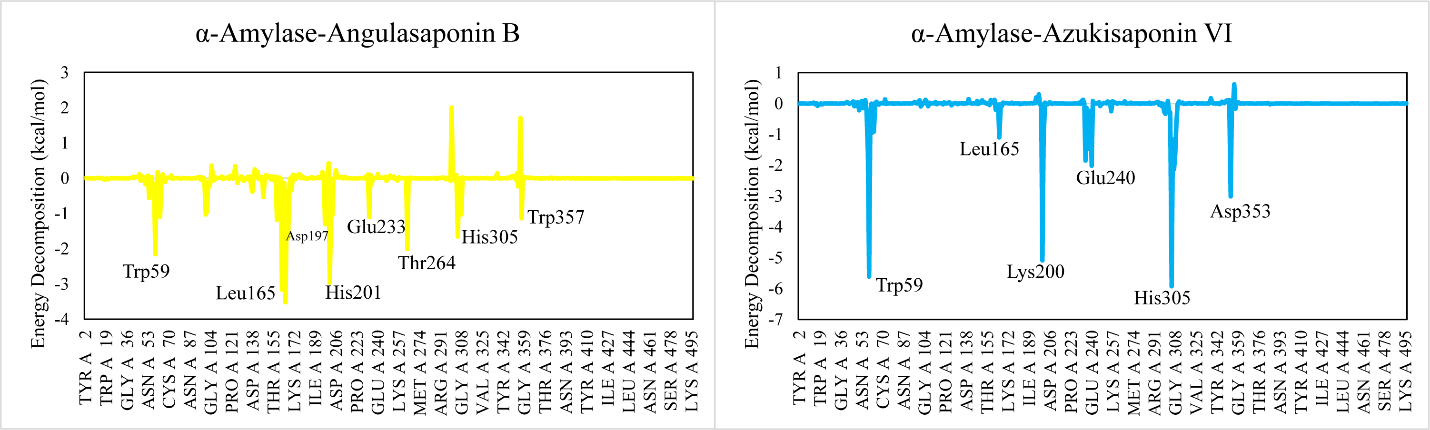


**Figure 8S**. The protein-ligand amino acid contacts of α-amylase complexes.


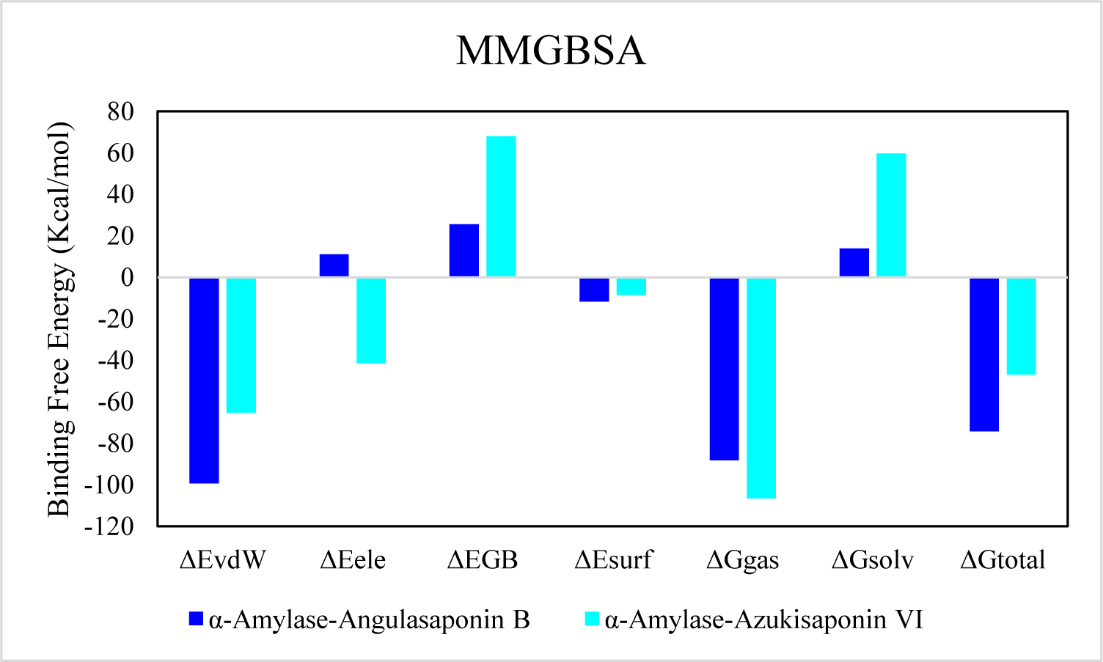
**Figure 9S**. The binding energy contribution comparison of the amylase complexes.

**
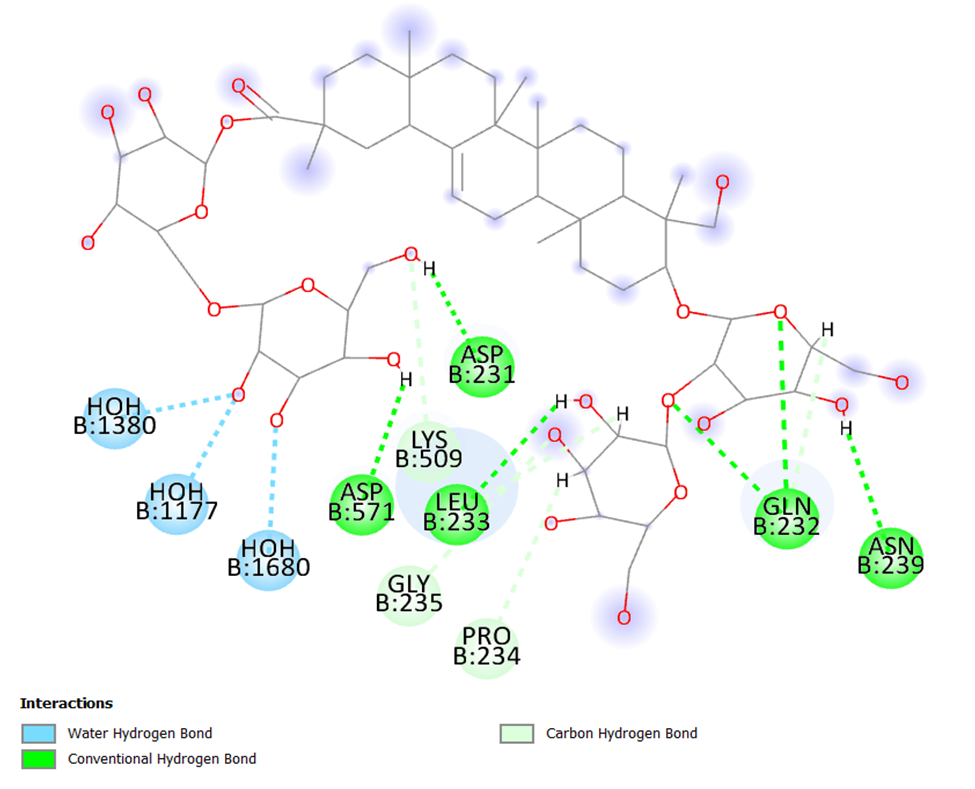
**

**Figure 10S**. 2D interactions of angulasaponin C in the binding pocket of sucrase enzyme

**
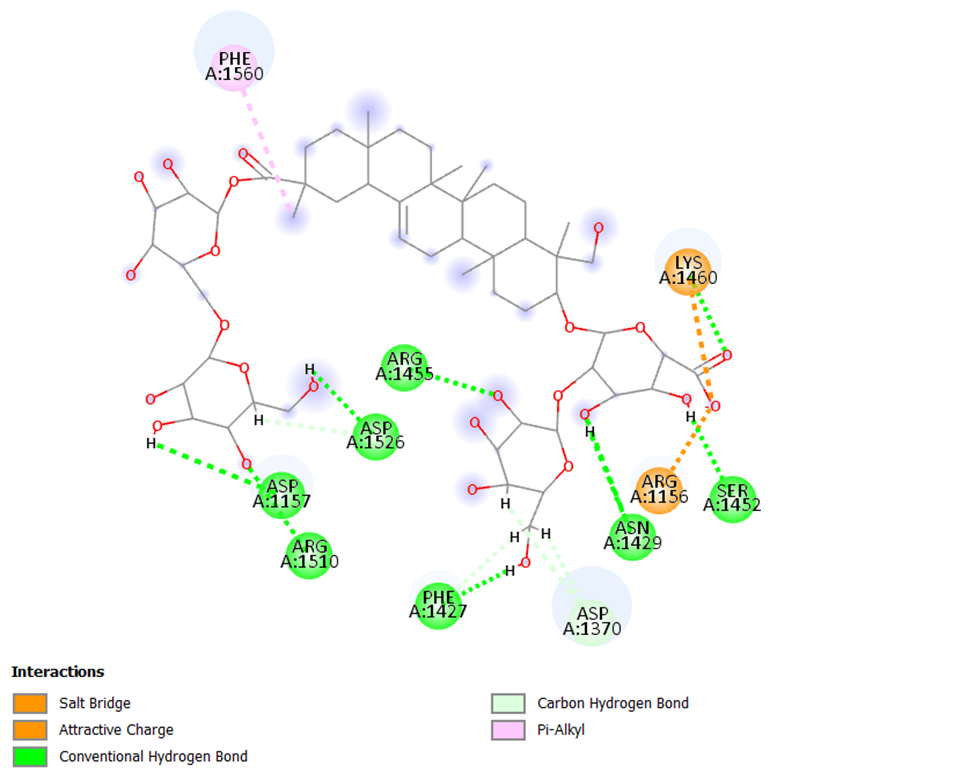
Figure 11S.** 2D interactions of angulasaponin C in the binding pocket of maltase enzyme.


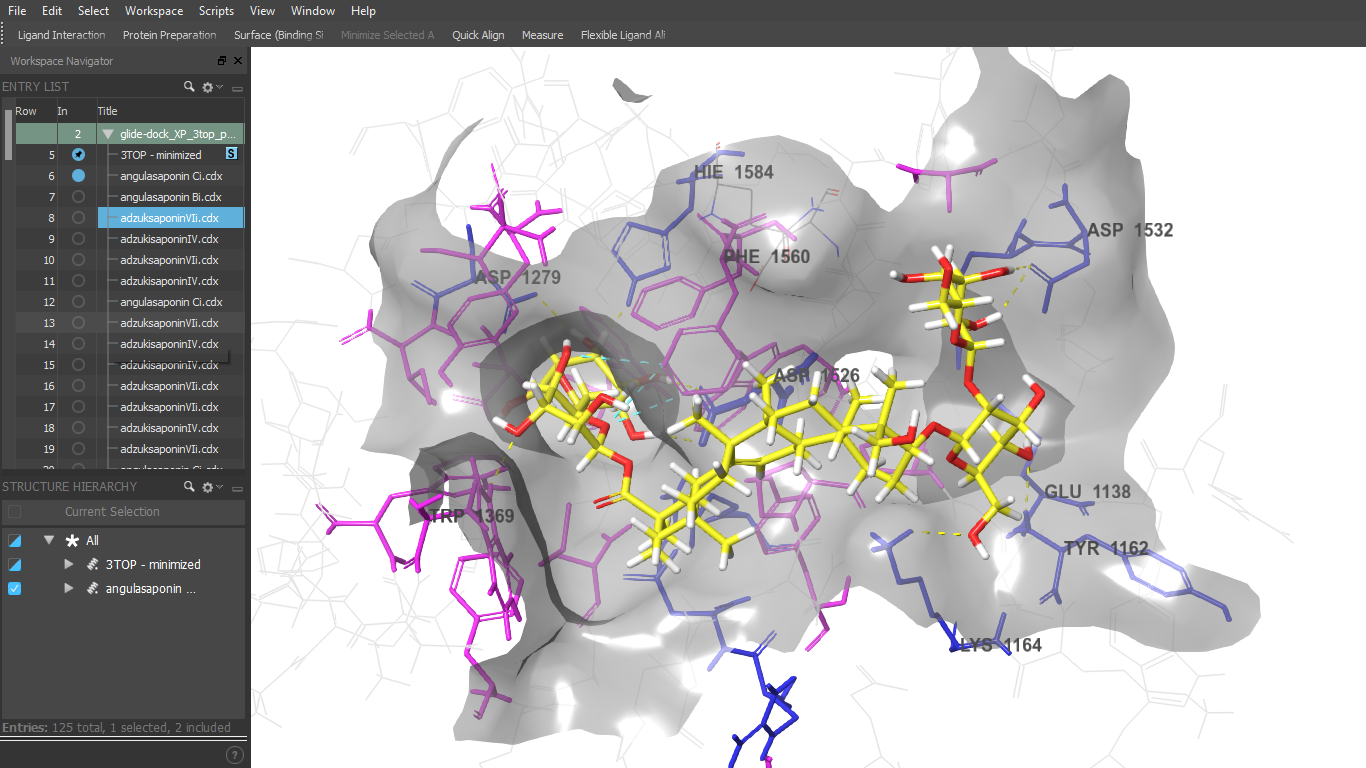


**Figure 12S**. Angulasaponin C (yellow) inside the MGAM-C binding site. Hydrophobic residues (Magenta), polar residues(blue)

**
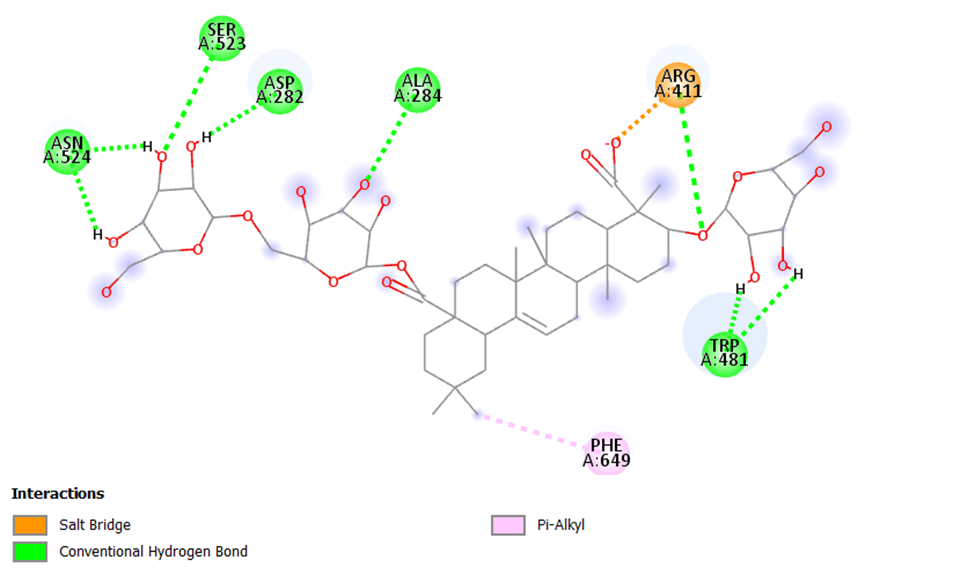

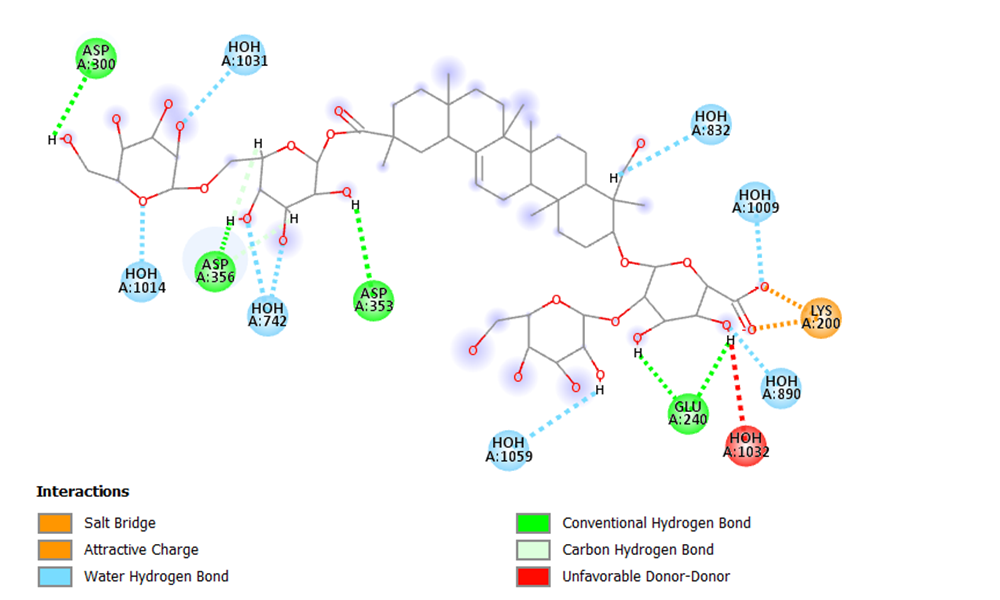
Figure 13S**. 2D interactions of adzukisaponin IV in the binding pocket of α-glucosidase

**Figure 14S**. 2D interactions of adzukisaponin VI in the binding pocket of α-amylase protein.


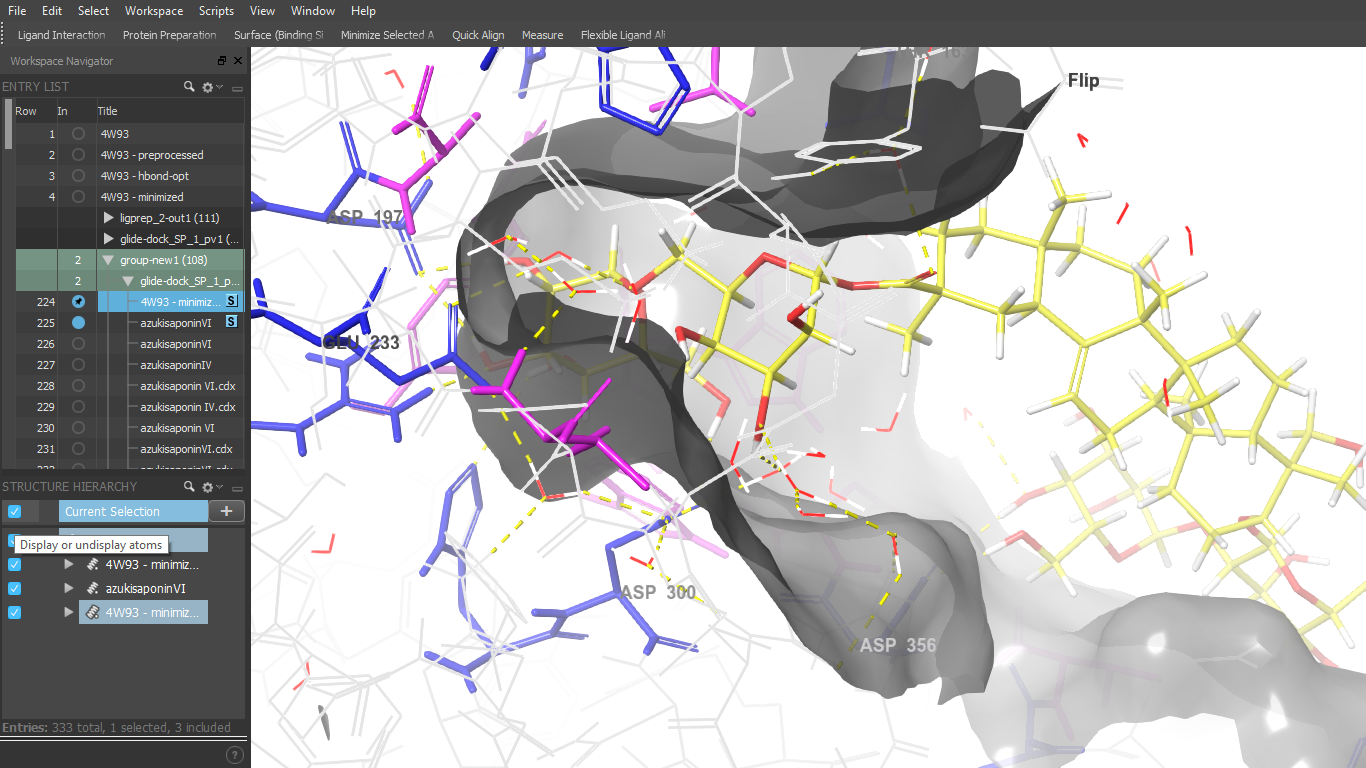
**Figure 15S**. Adzukisaponin VI in the binding site of 4w93

**
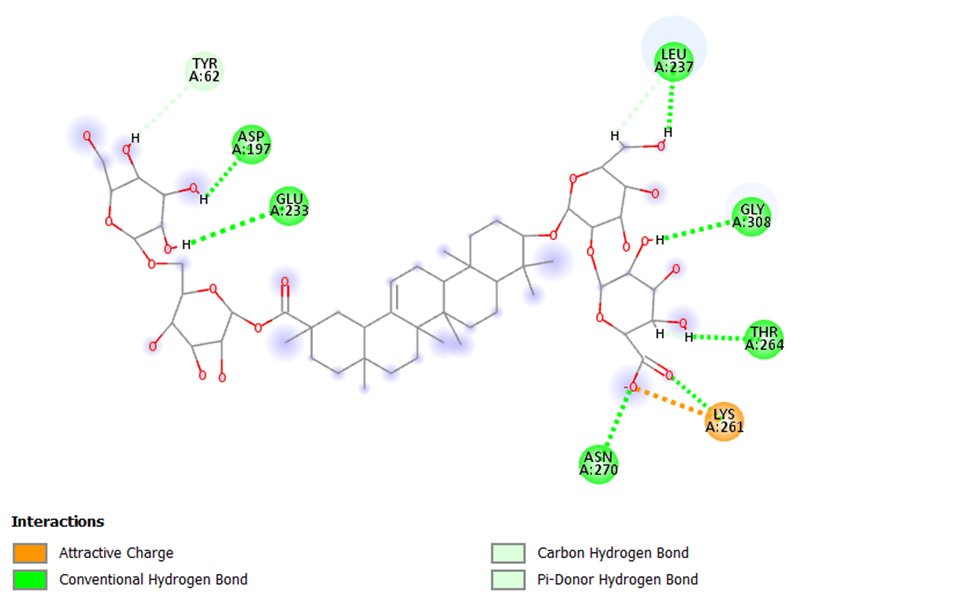
**

**Figure 16S**. 2D interactions of angulasaponin B in the binding site of α-amylase protein.
